# Efficient electroreduction of CO_2_ to C_2+_ products on CeO_2_ modified CuO†

**DOI:** 10.1039/d1sc01117k

**Published:** 2021-03-30

**Authors:** Xupeng Yan, Chunjun Chen, Yahui Wu, Shoujie Liu, Yizhen Chen, Rongjuan Feng, Jing Zhang, Buxing Han

**Affiliations:** Beijing National Laboratory for Molecular Sciences, CAS Key Laboratory of Colloid and Interface and Thermodynamics, CAS Research/Education Center for Excellence in Molecular Sciences, Institute of Chemistry, Chinese Academy of Sciences Beijing 100190 P. R. China Chenchunjun@iccas.ac.cn hanbx@iccas.ac.cn; University of Chinese Academy of Sciences Beijing 100049 China; Chemistry and Chemical Engineering of Guangdong Laboratory Shantou 515063 China; Physical Science Laboratory, Huairou National Comprehensive Science Center Beijing 101400 China; Shanghai Key Laboratory of Green Chemistry and Chemical Processes, School of Chemistry and Molecular Engineering, East China Normal University Shanghai 200062 China; Hefei National Laboratory for Physical Sciences at the Microscale, Key Laboratory of Strongly-Coupled Quantum Matter Physics of Chinese Academy of Sciences, National Synchrotron Radiation Laboratory, Key Laboratory of Surface and Interface Chemistry and Energy Catalysis of Anhui Higher Education Institutes, Department of Chemical Physics, University of Science and Technology of China 230026 Hefei Anhui People's Republic of China; Institute of High Energy Physics, Chinese Academy of Sciences Beijing 100049 China

## Abstract

Electrocatalytic reduction of CO_2_ into multicarbon (C_2+_) products powered by renewable electricity offers one promising method for CO_2_ utilization and promotes the storage of renewable energy under an ambient environment. However, there is still a dilemma in the manufacture of valuable C_2+_ products between balancing selectivity and activity. In this work, cerium oxides were combined with CuO (CeO_2_/CuO) and showed an outstanding catalytic performance for C_2+_ products. The faradaic efficiency of the C_2+_ products could reach 75.2% with a current density of 1.21 A cm^−2^. *In situ* experiments and density functional theory (DFT) calculations demonstrated that the interface between CeO_2_ and Cu and the subsurface Cu_2_O coexisted in CeO_2_/CuO during CO_2_RR and two competing pathways for C–C coupling were promoted separately, of which hydrogenation of *CO to *CHO is energetically favoured. In addition, the introduction of CeO_2_ also enhanced water activation, which could accelerate the formation rate of *CHO. Thus, the selectivity and activity for C_2+_ products over CeO_2_/CuO can be improved simultaneously.

## Abstract 2

CO_2_ can be efficiently converted into C_2+_ products on CeO_2_ modified CuO catalysts and the faradaic efficiency could reach 75.2% with a current density of 1.21 A cm^−2^.

## Graphical abstract

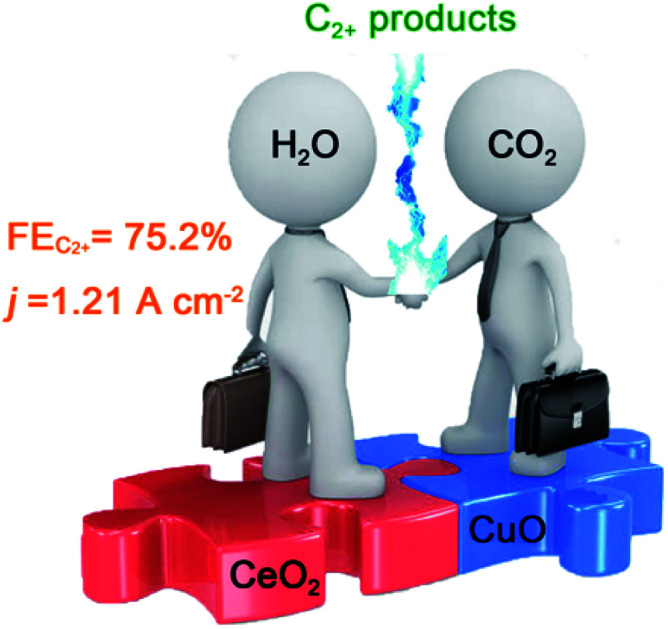

## Introduction

Conversion of CO_2_ into valuable chemicals using electrochemical methods provides a promising way to combat accumulated carbon emissions and also to store renewable energy.^1–6^ Continuous progress has been made in the field of the electrochemical CO_2_ reduction reaction (CO_2_RR), especially for monocarbon products like carbon monoxide (CO) and formate.^7–16^ However, the manufacture of valuable C_2+_ products in CO_2_RR, such as ethylene (C_2_H_4_), ethanol (C_2_H_5_OH) and *n*-propanol (*n*-C_3_H_7_OH), still has to balance selectivity and activity,^17–25^ which obstructs further industrial applications. To achieve a commercial current density (>100 mA cm^−2^) as well as high selectivity for C_2+_ products in CO_2_RR,^26–32^ highly efficient and robust electrocatalysts are required.

Cu-based catalysts are the most promising electrocatalysts for converting CO_2_ into C_2+_ products,^33–45^ owing to their moderate adsorption capacity for the crucial intermediate (*CO). Based on previous reports,^1,18,46,47^ the selectivity of C_2+_ products over Cu-based catalysts can be notably improved by the introduction of another component but the understanding of the structure–selectivity relationship remains controversial because the valence state and the microstructure of copper may be influenced simultaneously. What is more, complexity also exists in the production of C_2+_ products during CO_2_RR due to the C–C coupling step involved, which not only contains multiple electron-transfer and protonation steps,^6^ but also exhibits various potential coupling paths on heterogeneous catalysts. As a result, it is necessary to comprehensively reveal the role of another component in the promotion of the selectivity towards C_2+_ products during CO_2_RR.

Given the neutral or basic electrolyte used in CO_2_RR, H_2_O can serve as the hydrogen source and the activity should be bound up with the activation of H_2_O in CO_2_RR.^47^ According to the Sabatier principle, the energy barrier for the activation of water should be particularly controlled, which could provide enough hydrogen for the hydrogenation of intermediates but not cause excessive production of H_2_. Considering cerium oxide (CeO_2_) has a high activity for water activation in CO_2_ hydrogenation and shows poor activity for the hydrogen evolution reaction (HER),^48–50^ we can assume that the activity for C_2+_ products would be improved compared to the CeO_2_ modified Cu-based catalyst in CO_2_RR.

Herein, we used CeO_2_ to modify CuO to obtain CeO_2_/CuO catalysts, and both a high current density and selectivity towards C_2+_ products were achieved in CO_2_RR. A faradaic efficiency (FE) of 75.2% for the C_2+_ products could be attained on the catalyst with a total current density of 1.21 A cm^−2^ in a flow-cell system. The experiments and density functional theory (DFT) calculations indicate the energy of generation of *CHO is thermodynamically reduced by the interfacial effect compared to CeO_2_ modified CuO catalysts and the rapid activation of water around CeO_2_ accelerates the formation of *CHO kinetically, thus the C–C coupling step is facilitated *via* the *CHO route, endowing the CeO_2_/CuO catalyst with an excellent catalytic performance towards C_2+_ products.

## Results and discussion

**Fig. 1:**
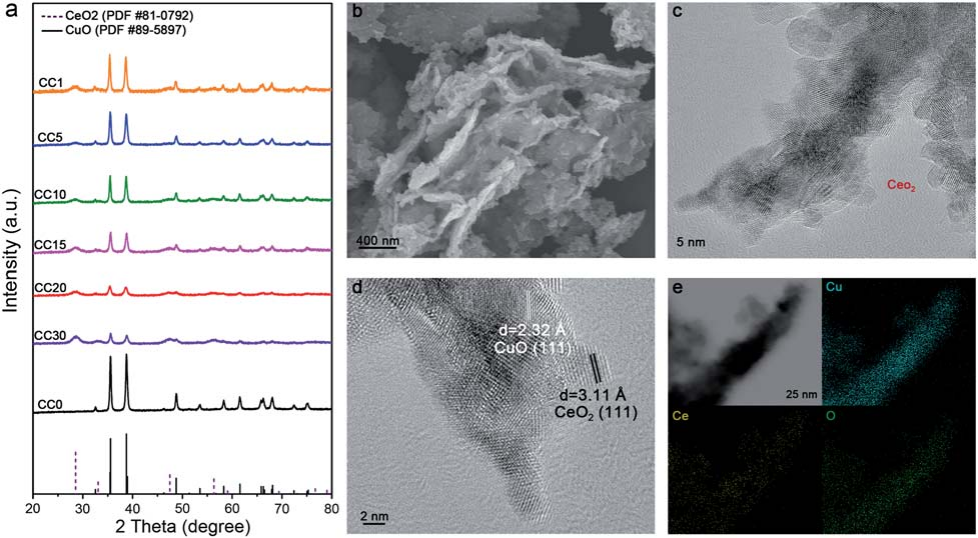
(a) The XRD patterns of the CC*X* composites with various Ce contents. (b and c) The SEM and TEM images of the CC20 (the red circle represents the CeO_2_ nanoparticles). (d) The HR-TEM image of the CC20. (e) The energy dispersive X-ray spectroscopy (EDS) maps of CC20.

The Ce(OH)_2_/Cu(OH)_2_ catalysts were first prepared by the coprecipitation method, then the CeO_2_/CuO catalysts were gained by annealing at 600 °C in air. As the amount of Ce in the catalysts increased from 0 to 30%, a set of peaks belonging to the CeO_2_ phase gradually emerged on the base of the primary CuO phase in the X-ray diffraction patterns (Fig. 1a), indicating the coexistence of CeO_2_ and CuO in the catalysts, and the CeO_2_/CuO catalysts were named CC*X* (*X* = the molar ratio of Ce and Cu times 100). From scanning electron microscopy (SEM) and transmission electron microscopy (TEM), we can observe that CeO_2_ nanoparticles below 5 nm were evenly loaded on the surface of CuO (Fig. 1b, c and S2†). Two typical *d*-spacings of 0.31 nm and 0.23 nm were observed in the image of high-resolution transmission electron microscopy (HR-TEM) for CC20 (Fig. 1d), corresponding to CeO_2_(111) and CuO(111). According to the distribution of the elements of Cu, Ce and O in the energy dispersive X-ray spectroscopy maps (Fig. 1e), the uniform element dispersion of Cu and Ce over the catalyst confirmed that CeO_2_ was uniformly dispersed on the CuO.

The electrocatalytic performance of the catalysts was evaluated in the flow cell and 1 M KOH was used as the electrolyte, as reported in our previous work.^51^ Before the CO_2_RR, the catalysts were firstly reduced around −0.3 V *vs.* RHE, which is more negative than the transformation of CuO to Cu (Fig. S3†). The polytetrafluoroethylene (PTFE) membrane (average pore size of 0.22 μm) was used as the gas diffusion electrode, and gaseous and liquid products were analyzed by gas chromatography (GC) and nuclear magnetic resonance (NMR) spectroscopy, respectively (Fig. S4†). The ^13^C labelled CO_2_ was used as the source of the reactant gas and the results verified that CO_2_ was the only carbon source in CO_2_RR (Fig. S5†).

**Fig. 2:**
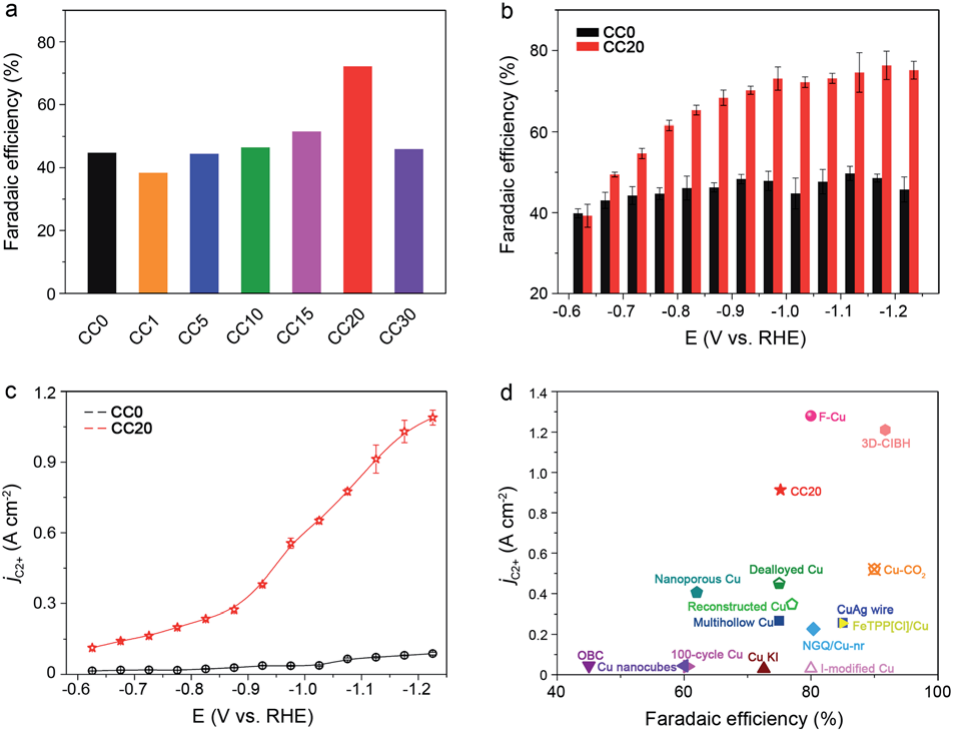
(a and b) The average FEs of C_2+_ products at various potentials in 1 M KOH over CC0 and CC20, respectively. (c) The partial current density of C_2+_ products at various potentials in 1 M KOH solution over CC0 and CC20. (d) A comparison of the average FEs and the current density of C_2+_ products on various reported catalysts and the literature sources are listed in the ESI (Table S1†).

Based on the performance of the CC*X* catalysts in CO_2_RR, a typical volcano plot between FE_C_2+__ and Ce content was observed at −1.02 V (*vs.* RHE) and CC20 exhibited the best performance at various applied potentials (Fig. 2a and S6–S8†). From the TEM (Fig. S2†), we can observe that the interfaces were produced with the increase of the Ce amount, thus we hypothesized that the selectivity of C_2+_ was related to the interfaces. However, for the CC30, the selectivity of C_2+_ products showed a significant decrease because too many Cu sites were covered by the CeO_2_. Thus, CC20 was chosen for further comparison with CC0. It can be clearly observed that CC20 showed outstanding efficiency for C_2+_ products in the CO_2_ reduction (Fig. 2b). The FE of C_2+_ products for CC20 could reach 75.2% at −1.12 V (*vs.* RHE), while that over CC0 was only 48.3% at the same condition. Moreover, the evolution of H_2_ was suppressed over CC20 and the FE of *n*-propanol was notably improved on CC20 compared to CC0 (Fig. S7–S9†), which might correlate with the escalation of C_2_ intermediates over CC20 (Fig. S9†). In the meantime, a significant increase was also achieved on the current density on CC20. It is very impressive that the partial current density of C_2+_ products (*j*_C_2+__) over CC20 could reach as high as 0.91 A cm^−2^ at −1.12 V (*vs.* RHE), which is about 10 times higher than that on CC0 (Fig. 2c). Compared with the state-of-the-art catalysts, the activity and selectivity for C_2+_ products over CC20 are among the highest values (Fig. 2d and Table S1†). The above results indicate that the introduction of CeO_2_ could significantly improve both the selectivity and activity for C_2+_ products. Moreover, the performance of CeO_2_ was also characterized (Fig. S12†), and only trace CO was detected at −0.87 V and −0.97 V (*vs.* RHE), while the current density was below 20 mA cm^−2^ at the applied potentials, indicating that pure CeO_2_ showed poor activity for CO_2_RR. Besides, the catalysts were characterized after the reaction and no obvious change was observed in the TEM images and XRD patterns (Fig. S14–S17†). As a result, the proper content of CeO_2_ would obviously benefit the catalytic performance of the CuO catalyst towards C_2+_ products in CO_2_RR.

To reveal the reasons for the superior catalytic performance of CC20 in the CO_2_RR, the electrochemical active surface areas (ECSAs) and electrochemical impedance spectroscopy (EIS) of the catalysts were studied. We can observe that similar ECSAs were obtained over the CC*X* catalysts with different CeO_2_ contents (Fig. S18†), indicating the similar surface area of the catalysts at the electrochemical conditions. Moreover, the charge transfer resistance (*R*_ct_) for the catalysts was also similar (Fig. S19†), suggesting that the discrepancy of the efficiency for C_2+_ products did not mainly originate from the slight difference of the ECSAs and electronic conductivity.

**Fig. 3:**
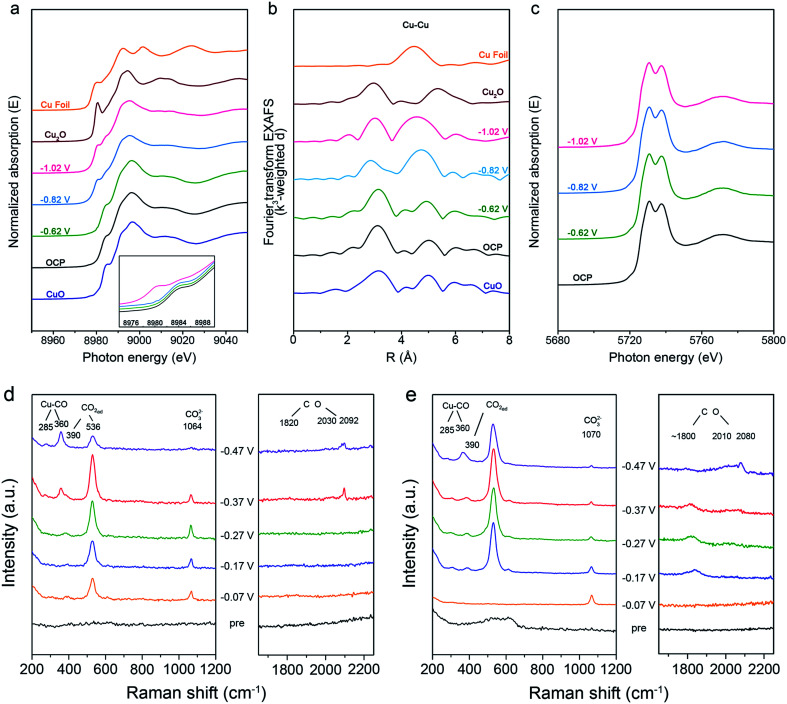
(a and b) *Operando* XANES and the corresponding Fourier transforms of *k*^3^-weighted EXAFS data at the Cu K-edge at various applied potentials (*vs.* RHE) over CC20 during CO_2_RR. (c) *Operando* XANES at the Ce L_3_-edge at various applied potentials (*vs.* RHE) over CC20 during CO_2_RR. (d) The *in situ* surface-enhanced Raman spectra for CC0 at various potentials (*vs.* RHE) during CO_2_RR. (e) The *in situ* surface-enhanced Raman spectra for CC20 at various potentials (*vs.* RHE) during CO_2_RR.

The catalytic performance of Cu-based catalysts was closely related to the oxidation state and local structure, which could alter the adsorption of intermediates,^30,41,42,52^ thus the *operando* X-ray absorption spectroscopy (XAS) was used to track the evolution of the oxidation state and local structure of Cu and Ce over CC0 and CC20 during CO_2_RR. At the open circuit potential (OCP), near the Cu K-edge, both the X-ray absorption near edge structure (XANES) and the *k*^3^-weighted Fourier-transformed (FT) extended X-ray absorption fine structure (EXAFS) spectra of CC0 and CC20 showed the typical features of CuO (Fig. 3a and b, S20 and S21†), indicating that CuO was dominant in CC0 and CC20 before the reaction. As the potential was applied as −0.62 V (*vs.* RHE), there was no obvious change in either the XANES or FT-EXAFS spectra. Meanwhile, CC0 and CC20 showed a low FE_C_2+__ and these results could be due to the large proportion of Cu(ii). When the applied potential decreased to −0.82 V (*vs.* RHE), features of Cu with low oxidation states emerged in the XANES spectra and EXAFS analysis also displayed that the Cu first shell coordination switched to the mixture of different Cu species over CC0 and CC20. It can be found that the FEs of C_2+_ at −0.82 V (*vs.* RHE) also showed a significant increase compared to that at −0.62 V (*vs.* RHE), suggesting the potential correlation between the Cu oxidation state and the FE_C_2+__ in CO_2_RR. Furthermore, according to the XANES spectra, the oxidation state of Cu in the catalysts continued to decrease and the results in the EXAFS data were different from the initial CuO-like state, demonstrating that Cu with a low oxidation state became the main phase. Interestingly, the FE of C_2+_ in CO_2_RR still slightly increased from −0.82 V (*vs.* RHE) to −1.02 V (*vs.* RHE) on CC0 and CC20, which supported the conclusion that the low-valent Cu species on catalysts were the active phase in CO_2_RR. Moreover, according to the distribution of various Cu species for CC20 and CC0 (Fig. S22 and S23†), we can observe that the Cu_2_O species occupied the higher proportion over CC20 than on CC0. These results indicated that the introduction of CeO_2_ could stabilize the Cu_2_O, which could be attributed to the interaction between Ce and Cu, and the role of Cu_2_O in CO_2_RR will be discussed in the later section. In addition, the *operando* XANES data at the Ce L_3_-edge of CC20 showed a negligible change during CO_2_RR (Fig. 3c), indicating that CeO_2_ remained stable during CO_2_RR.

Generally, the activity and selectivity of C_2+_ products are closely related to the surface species on the catalysts during the reduction. So, an *in situ* surface enhanced Raman spectroscopy (SERS) study was carried out to explore the surface species over CC0 and CC20 (Fig. 3d and e, S24†). After the pre-electrolysis at N_2_ atmosphere, only two weak bands at 524 and 610 cm^−1^ were observed, which belonged to Cu_2_O,^53^ and then disappeared in CO_2_ electrolysis. Instead, bands at 390 and 536 cm^−1^ emerged at negative potentials in CO_2_ electrolysis, which were attributed to the chemisorption of CO_2_ on the surface Cu.^57,58^ Furthermore, we can observe that no Cu_2_O could be found on both CC0 and CC20 during CO_2_RR from the Raman spectra. Combined with the results of the *operando* XAFS, we can assume that the Cu_2_O species exists on the subsurface of the catalysts, due to the Raman spectroscopy being sensitive to the surface species of the catalyst,^56,57^ which is consistent with previous reports.^21,59^ In addition, the signals of CeO_2_ cannot be found on CC20 in the Raman spectroscopy, this may be due to the signals of CeO_2_ being too weak under the existence of the electrolyte in the *in situ* experiments.^60^

As the applied potential negatively moved, both on CC0 and CC20, peaks at 285, 365, 1800–1860 and 2000–2100 cm^−1^ became cognizable, corresponding to the restricted rotation of adsorbed *CO on Cu, Cu–CO stretching, and bridge and top C≡O stretching, respectively.^54–58,61^ It is interesting to note that there was a distinct disparity in the performance of the above *CO related peaks over CC0 and CC20. For CC0, at −0.37 V (*vs.* RHE), *CO related peaks began to be observed and the peak around 1820 cm^−1^ was weak. On the contrary, those peaks were clearly present over CC20 after −0.17 V (*vs.* RHE), and the peak between 1800–1860 cm^−1^ even showed a red shift while the peak at 2000–2100 cm^−1^ became strong. The difference between those two catalysts supported the conclusion that CO_2_ could be transformed into CO at lower applied potentials on CC20 than on CC0, indicating the superior activity of CC20 towards CO in CO_2_RR, which is consistent with the results in the electrochemical tests (Fig. S11†). Moreover, the excellent catalytic capability for the CO product in CO_2_RR should be favourable for the following steps in CO_2_RR.

**Fig. 4:**
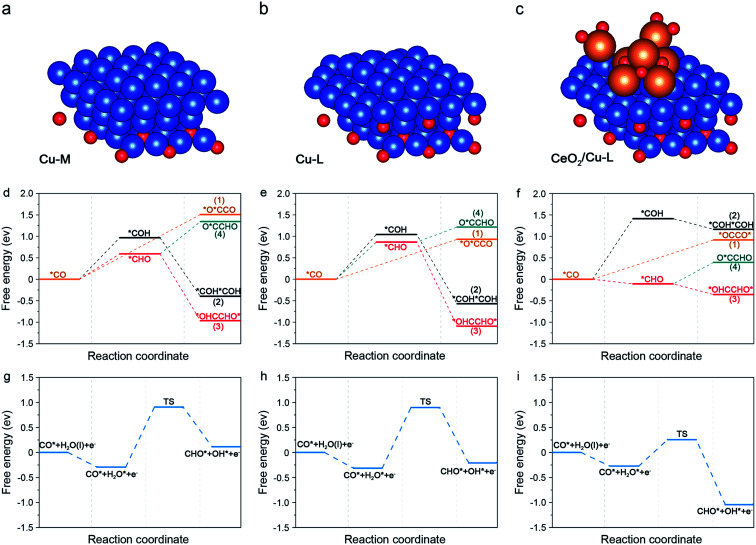
(a–c) The side views of Cu-M, Cu-L and CeO_2_/Cu-L, in which the blue balls, red balls and orange balls stand for Cu, oxygen, and carbon and hydrogen, respectively. (d–f) The reaction energy diagram for the CO_2_RR to describe the possible C–C coupling step from *CO on Cu-M, Cu-L and CeO_2_/Cu-L. (g–i) The reaction energy diagram for *CO hydrogenation to *COH on Cu-M, Cu-L and CeO_2_/Cu-L, respectively.

DFT calculations were then performed to elucidate the mechanism of the crucial C–C coupling step and to gain insight into the excellent performance of CC20 in CO_2_RR. According to the above results, the introduction of CeO_2_ can not only form the interface between CeO_2_ and Cu, but can also stabilize the subsurface Cu_2_O. Although both the interface and subsurface Cu_2_O can promote the CO_2_RR,^62,63^ they have been studied separately in previous reports.^64,65^ Thus, the role of interface and subsurface Cu_2_O on enhancing the C_2+_ products should be studied simultaneously and three specific models were used to study the effect of subsurface Cu_2_O and CeO_2_ on promoting C–C coupling (Fig. S27†). First, a model with more metallic Cu on the surface and less Cu_2_O on the subsurface (Cu-M) was built to represent the CC0 (Fig. 4a). Then, a model with less metallic Cu on the surface and more Cu_2_O on the subsurface (Cu-L) was built to represent the CC20 without CeO_2_ (Fig. 4b). Last, Cu-L with CeO_2_ on the surface (CeO_2_/Cu-L) was built to represent CC20 (Fig. 4c). The Cu(111) and CeO_2_(111) were chosen as the basic models according to the results of XRD (Fig. S16†), and the ratio of Cu and Cu_2_O was set according to the results of *in situ* XAS (Fig. S22 and S23†).

Generally, CO_2_ can be first reduced into CO through the *COOH pathway,^28^ and the adsorbed CO is regarded as the common intermediate for the C_2+_ products in CO_2_RR.^66^ In this condition, four potential reaction pathways are taken into account in the C–C coupling step and all of them are generated from the vital intermediate *CO (Fig. S28–S33†).

1 *CO + *CO → *CO–*CO

2 *CO + H^+^ + e^−^ → *COH, *COH + *COH → *COH–*COH

3 *CO + H^+^ + e^−^ → *CHO, *CHO + *CHO → *CHO–*CHO

4 *CO + H^+^ + e^−^ → *CHO, *CHO + *CO → *CO–*CHO

On Cu-M, the energy of 1.51 eV is required for the dimerization of *CO (path 1), higher than that on Cu-L (0.93 eV), indicating that more subsurface Cu_2_O are beneficial for the C–C coupling through the *CO–*CO route (Fig. 4d and e), which is consistent with previous reports.^21,63^ Further addition of CeO_2_ on Cu-L barely alters the energy for the dimerization of *CO (0.92 eV) compared with Cu-L (Fig. 4f). Consequently, we can assume that the energy for dimerization of *CO can be decreased by subsurface Cu_2_O, however, the energy for the formation of the *O*CCO intermediate was still very high, indicating that C–C coupling through *CO dimerization is difficult.

We notice that both path 2 and path 3 suffer from the endothermic protonation of adsorbed *CO and the subsequent exothermic coupling step in all the models. In terms of the lower energy needed for the generation of *CHO compared to *COH, we can assume that the C–C coupling step would prefer the *CHO route rather than the *COH route. However, the formation of *CHO in each model is different in energy. 0.59 eV is required for the hydrogenation of *CO into *CHO on Cu-M, while a higher energy of 0.87 eV is needed on Cu-L, suggesting that more subsurface Cu_2_O were not advantageous for the formation of *CHO. This may be due to the fact that the adsorption of *CHO can be affected by the subsurface Cu_2_O, and the intrinsic reason should be further studied. Surprisingly, the energy for the hydrogenation of *CO into *CHO dramatically declined to −0.11 eV and became exothermic near the interface of CeO_2_ and Cu-L (Fig. S32 and S33†). The above results convincingly demonstrate that *CHO is easily formed from *CO on CC20 and this should be attributed to the introduction of CeO_2_ and the formed interface, rather than more subsurface Cu_2_O. Furthermore, for the following C–C coupling step related to *CHO, the coupling of *CO and *CHO (path 4) is also possible in theory except for the dimerization of *CHO (path 3). Nevertheless, the coupling of *CO and *CHO is endoenergic over all surfaces, suggesting that the exoenergic dimerization of *CHO would be favourable to the coupling process. On the whole, the coupling of *CHO into *OHCCHO* is most favourable in the C–C coupling step among the above possible pathways in the three models and the process even becomes spontaneous in the presence of CeO_2_. Ma and co-workers also found that the coupling between *CHO showed lower barriers on the Cu(111) surfaces.^28^ In consequence, the *CHO route (path 3) is favoured on the three models in the C–C coupling step and becomes exothermal on CC20 due to the formed interface, elucidating the high FE for C_2+_ products on CC20. In addition, we can observe that all the intermediates were mainly adsorbed on the exposed Cu sites, so we can assume that Cu was the active site.

In consideration of the 1 M KOH used in CO_2_RR, H_2_O should be considered as the hydrogen donor. As a result, we introduced the water activation process to further study the kinetic process for the formation of *CHO. For Cu-M and Cu-L, water is spontaneously adsorbed on the surface Cu and then the high energy barriers of 1.20 eV and 1.21 eV are needed to form the transient state (TS) for the following formation of *CHO (Fig. 4g, h and S34†), respectively. For the CeO_2_/Cu-L, H_2_O would like to be adsorbed around the CeO_2_ and undergo dissolution to offer active hydrogen. Due to the sufficient active hydrogen, the barrier for TS decreases to only 0.53 eV (Fig. 4i), making the formation of *CHO more kinetically feasible on CC20. In conclusion, the formation of *CHO is faster on CeO_2_/Cu-L than that on other surfaces without CeO_2_ due to the rapid water activation around CeO_2_, which agrees with the high current density for CC20 during CO_2_RR.

In addition, the DFT calculations were also carried out at the bias of −0.5 V and −1.12 V (Fig. S35 and S36†), respectively, which are the requirement to overcome the C–C coupling step and are consistent with the reaction potential. At the selected potentials, we can observe that hydrogenation of *CO to *CHO and then coupling of *CHO into *CHO–*CHO still remain the favourable path for C–C coupling on each surface during CO_2_RR. More importantly, both the thermodynamic process and kinetic process for the formation of *CHO on CeO_2_/Cu-L are more feasible than that on Cu-M or Cu-L. These results elucidate the motivation for the simultaneously enhanced selectivity and activity for C_2+_ products by the introduction of CeO_2_.

According to the DFT calculation results, hydrogenation of *CO to *CHO played a crucial role for enhancing the C_2+_ products, especially incorporated with the activation of H_2_O. In consequence, the kinetic isotopic effects (KIEs) of H/D over CC0 and CC20 were measured to further ensure the role of water activation in CO_2_RR (Fig. S37†). As the H_2_O was replaced by D_2_O as the solvent in 1 M KOH solution, the formation rate of ethylene significantly decreased on CC0, and the KIE (the ratio of ethylene formation rates in H_2_O and D_2_O) was about 2.0, which suggests that dissolution of H_2_O should be involved in the rate-determining step (RDS) for the ethylene formation. On the contrary, the KIE value on CC20 was nearly 1, suggesting that hydrogen was not related to the rate-determining step over CC20. The above confirmed the results of the DFT calculations that the *CHO route was endothermic on CC0 and exothermic on CC20. In addition, CC20 yielded 312 mA cm^−2^ at −1.12 V (*vs.* RHE) for HER under N_2_ atmosphere, about 2.5 times higher than that on CC0 (Fig. S37†), supporting the argument that CC20 had a superior capability for water activation. Thus, it can be concluded that the existence of CeO_2_ accelerated the dissolution of H_2_O to offer enough active hydrogen and thus benefited the generation of *CHO, which enhances the C–C coupling step through the dimerization of *CHO.

## Conclusions

In conclusion, the introduction of CeO_2_ on the surface of CuO significantly enhanced the selectivity and activity towards C_2+_ products in CO_2_RR. Experimental and *in situ* SERS results confirmed the generation of the important intermediate CO was notably enhanced on CC20, which offered abundant precursors for the following steps. More importantly, DFT calculations revealed that the C–C coupling step followed the *CHO route and was facilitated both thermodynamically and kinetically on CC20 by the interfacial effects and the rapid water activation, respectively, findings which were also supported by the KIE experiments. Consequently, the FE of the C_2+_ products could reach up to 75.2% with the current density of 1.21 A cm^−2^ at −1.12 V (*vs.* RHE) in 1 M KOH. We believe that the findings in this work contribute to understanding the role of the introduced component and could help to design efficient catalysts towards C_2+_ products in CO_2_RR.

## Author contributions

X. P. Y., C. J. C. and B. X. H. proposed the project, designed the experiments and wrote the manuscript; X.P. Y. performed the whole experiments; Y. H. W., S. J. L., Y. Z. C., R. J. F. and J. Z. assisted in analyzing the experimental data; B. X. H. supervised the whole project.

## Conflicts of interest

There are no conflicts to declare.

## Supplementary Material

SC-012-D1SC01117K-s001
