# Supplementary material for: Efficient electroreduction of CO2 to C2+ products on CeO2 modified CuO
Source: Chem Sci. 2021 Mar 30;12(19):6638–45. doi: 10.1039/d1sc01117k (PMC8132937; doi:10.1039/d1sc01117k)
Supplement: SC-012-D1SC01117K-s001 [file SC-012-D1SC01117K-s001.pdf]

Electronic Supplementary Material (ESI) for Chemical Science.
This journal is © The Royal Society of Chemistry 2021

Efficient Electroreduction of CO_2 to C_{2+} Products on CeO_2 modified CuO

Xupeng Yan,[a, b] Chunjun Chen,[a, b] Yahui Wu,[a, b] Shoujie Liu,[c] Yizhen Chen,[f] Rongjuan Feng,[a] Jing Zhang,[g] and Buxing Han[a, b, d, e]*

a Beijing National Laboratory for Molecular Sciences, Key Laboratory of Colloid and Interface and Thermodynamics, Institute of Chemistry, Chinese Academy of Sciences, Beijing 100190, P. R. China.

b School of Chemistry and Chemical Engineering, University of Chinese Academy of Sciences, Beijing 100049, P. R. China.

c Chemistry and Chemical Engineering of Guangdong Laboratory, Shantou 515063, China.

d Physical Science Laboratory, Huairou National Comprehensive Science Center, No. 5 Yanqi East Second Street, Beijing 101400, P. R. China.

e Shanghai Key Laboratory of Green Chemistry and Chemical Processes, School of Chemistry and Molecular Engineering, East China Normal University, Shanghai 200062, P. R. China.

f National Laboratory for Physical Sciences at the Microscale, Key Laboratory of Strongly-Coupled Quantum Matter Physics of Chinese Academy of Sciences, National Synchrotron Radiation Laboratory, Key Laboratory of Surface and Interface Chemistry and Energy Catalysis of Anhui Higher Education Institutes, Department of Chemical Physics, University of Science and Technology of China, 230026 Hefei, Anhui, People's Republic of China.

g Institute of High Energy Physics, Chinese Academy of Sciences, Beijing 100049, China.

Experimental Section

Chemicals and materials： Cerium(III) Nitrate ($Ce(NO_3)_3$, ≥ 99.0%), Copper nitrate trihydrate ($Cu(NO_3)_2 \cdot 3H_20$, ≥ 99.0%), potassium hydroxide (KOH, ≥ 85%) and sodium 2, 2-dimethyl-2-silapentane-5-sulfonate (DSS, 99%) were purchased from Sigma-Aldrich. Ammonium hydroxide ($NH_3 \cdot H_2O$, 65% ~ 68%), sodium hydroxide (NaOH, ≥ 99.0%) and Ni foam were purchased from Sinopharm Chemical Reagent Co. Ltd. D_2O (98%). Ni foam was purchased from Beijing Innochem Science & Technology Co. Ltd. All the chemicals were used as received. N_2 (99.999%) and CO_2 (99.999%) were provided by Beijing Analytical Instrument Company. Deionized water was used in the experiments.

Synthetic procedures for CCX and CeO_2. CC0 was synthesized in a facile way according to a literature method.[1] First, 1 g $Cu(NO_3)_2 \cdot 3H_2O$ were dissolved in 100 mL deionized water, followed by the addition of 30 mL 0.15 M $NH_3 \cdot H_2O$ to obtain the blue solution with a constant stirring. Then about 8 mL 1 M NaOH solution was dropped into the solution to adjust the pH value to 10, and kept stirring for 30 minutes. After centrifugation, precipitates were collected and dried in a vacuum freeze drying oven for 24 h. The dried samples were then annealed at 600 ºC for 2 h, while the temperature was rapidly risen from ambient temperature to 600 ºC in 18 minutes. The different CCX were prepared by the same method except $Cu(NO_3)_2 \cdot 3H_2O$ is replaced by the mix $Ce(NO_3)_3$ and $Cu(NO_3)_2 \cdot 3H_2O$ (the molar ratios of Ce and Cu were 1%, 5%, 10% 15%, 20% and 30%). The CeO_2 was prepared by the same method except $Cu(NO_3)_2 \cdot 3H_2O$ is replaced by $Ce(NO_3)_3$.

Characterization of the materials. The SEM and TEM characterizations were carried out using a HITACHI S-4800 and JEOL JEM-2100Fequipped with EDS, respectively. The operando X-ray adsorption spectroscopy (XAS) measurements were performed using a modified flow cell at the 1W1B, 1W2B beamline at Beijing Synchrotron Radiation Facility (BSRF).

Preparation of electrodes. To construct the cathode electrode, a catalyst slurry that contained 5 mg of obtained catalysts, 1 mL of methanol and 20 μL of Nafion ionomer solution (5 wt% in H_2O) was first mixed and sonicated for 30 min. Then, the catalyst slurry (0.2 mL) was slowly drop cast onto a PTFE membrane (Fuel Cell Store) under vacuum to achieve a catalyst loading of ~1.0 mg cm^{-2}. Ni foam were used anode electrode.

Electrochemical study. Electrochemical studies were conducted in an electrochemical flow cell which including a gas chamber, a cathodic chamber, and an anodic chamber, as reported in our previous work.[2] An anion exchange membrane (FumasepFAA-3-PK-130) was used to separate the anodic and cathodic chambers, and an Ag/AgCl electrode and Ni foam were used as the reference and counter electrodes, respectively. The electrolysis was

conducted using a CHI-660e electrochemical workstation equipped with a high current amplifier CHI 680c. The measured potentials after *iR* compensation (3.6 ohm was used) were rescaled to the RHE by *E* (versus RHE) = *E* (versus Ag/AgCl) + 0.209 V + 0.0591V/pH × pH. For performance studies, 1 M KOH was used as the electrolyte, and it was circulated through the cathodic and anodic chambers using peristaltic pumps at a rate of 20 mL min^{-1}. The flow rate of CO_2 gas through the gas chamber was controlled to be 20 sccm using a digital gas flow controller. The KIE experiment was conducted in the 1 M KOH solution, in which H_2O was replaced by D_2O.

Product analysis. The gaseous product of electrochemical experiments was collected using a gas bag (the first bag was collected from the 10th minute at every applied potential and each bag was collected for 15 minutes) and analyzed by gas chromatography (GC, HP 4890D), which was equipped with TCD detector using argon as the carrier gas. The liquid product was analyzed by ^{1}H NMR (Bruker Advance III 400 HD spectrometer) in deuteroxide.

Calculations of Faradaic efficiencies of gaseous and liquid products

Calculations of Faradaic efficiencies of Liquid products: After electrolysis, a certain amount of internal standard solution was added to the electrolyte as the internal standard. Because the concentration of internal standard was known, the moles of liquid products can be calculated from integral areas and calibration curves. To accurately integrate the products in NMR analysis, two standards located in different regions were used in NMR analysis. The sodium 2, 2-dimethyl-2-silapentane-5-sulfonate (DSS) was the reference for n-propanol, ethanol and acetic acid, and the phenol was the reference for formate. 400 μL catholyte after the reaction was mixed with 100 μL 6 mM DSS solution, 100 μL 200 mM phenol and 200 μL D_2O, and then analyzed by ^{1}H NMR (Bruker Advance III 400 HD spectrometer).

The Faradaic efficiency of liquid product is:

$$\boldsymbol{FE}=\frac{\boldsymbol{moles\ of\ product}}{\boldsymbol{Q/nF}}\times \mathbf{100\%}$$

(Q: charge (C); F：Faradaic constant (96485 C/mol); n: the number of electrons required to generate the product)

Calculations of Faradaic efficiencies of Gaseous products. From the GC peak areas and calibration curves for the TCD detector, we can obtain the V % of gaseous products . Since the flow rate of the outlet was monitored to be constant, the moles of gaseous products can be calculated. The Faradaic efficiency of gaseous product is:

$$\boldsymbol{FE}=\frac{\boldsymbol{moles\ of\ product}}{\boldsymbol{Q/nF}}\times \mathbf{100\%}$$

(Q: charge (C); F：Faradaic constant (96485 C/mol); n: the number of electrons required to generate the product)

Electrochemical impedance spectroscopy (EIS) study. The EIS measurement was carried out in 1 M KOH at an open circuit potential (OCP) with an amplitude of 5 mV of 10^{-1} to 10^6 Hz.

Double-layer capacitance (C_{dl}) measurements. The catalyst was dropped on the 6mm glass carbon electrode (GCE) as the work electrode. The electrochemical active surface area is proportional to C_{dl} value. C_{dl} was determined by measuring the capacitive current associated with double-layer charging from the scan-rate dependence of cyclic voltammogram (CV). The CV ranged from -0.43 V to -0.53 V vs. RHE. The C_{dl} was estimated by plotting the Δj (j_a-j_c) at -0.48 V vs. RHE against the scan rates, in which the j_a and j_c are the anodic and cathodic current density, respectively. The scan rates were 20, 30, 40, 50, 60, 80, 100 and 120 mV s^{-1}.

***In-situ* Raman measurements.** *In-situ* Raman measurements were carried out using a Horiba LabRAM HR Evolution Raman microscope in a modified flow cell (purchased from Gaossunion Technology Co., Ltd.). A 785-nm laser was used and signals were recorded using a 20 s integration and by averaging two scans. The catalysts were firstly detected as the "pre" line in Fig. 3d and 3E to confirm the structure properties after the pre-electrolysis, and the corresponding lines were amplified to exhibit the signal of Cu_2O. The *iR* compensation was conducted in the *operando* XAS experiment at each potential, and a 15-minute electrolysis was conducted to gain the steady state before the collection of Raman spectra with constantly flowed gaseous CO_2.

XAFS measurements

The X-ray absorption find structure spectra data (Fe K-edge) were collected at 1W1B station in Beijing Synchrotron Radiation Facility (BSRF, operated at 2.5 GeV with a maximum current of 250 mA). The data were collected in fluorescence excitation mode using a Lytle detector. All samples were pelletized as disks of 13 mm diameter with 1mm thickness using graphite powder as a binder.

Operando XAS measurements were conducted in a custom-designed flow cell as our previous reports.[2] Catalysts were filtered on the PTFE membrane as the working electrode and 1 M KOH was used as the electrolyte. The saturated KCl Ag/AgCl electrode was chosen as the reference electrode and the Ni foam was used as the counter electrode. The *iR* compensation was conducted in the *operando* XAS experiment at each potential, and a 15-minute electrolysis was conducted to gain the steady state before the collection of XAS spectra with constantly flowed gaseous CO_2.

The acquired EXAFS data were processed according to the standard procedures using the Athena and Artemis implemented in the IFEFFIT software packages. The fitting detail is described below:

The acquired EXAFS data were processed according to the standard procedures using the ATHENA module implemented in the IFEFFIT software packages. The EXAFS spectra were obtained by subtracting the post-edge background from the overall absorption and then normalizing with respect to the edge-jump step. Subsequently, the $\chi(k)$ data of were Fourier transformed to real (R) space using a hanning windows (dk=1.0 $Å^{-1}$) to separate the EXAFS contributions from different coordination shells. To obtain the quantitative structural parameters around central

atoms, least-squares curve parameter fitting was performed using the ARTEMIS module of IFEFFIT software packages.

The following EXAFS equation was used:

$$\chi(k) = \sum_j \frac{N_j S_o^2 F_j(k)}{k R_j^2} \exp[-2k^2\sigma_j^2] \exp[\frac{-2R_j}{\lambda(k)}] \sin[2kR_j + \phi_j(k)]$$

S_0^2 is the amplitude reduction factor, $F_j(k)$ is the effective curved-wave backscattering amplitude, N_j is the number of neighbors in the jth atomic shell, R_j is the distance between the X-ray absorbing central atom and the atoms in the jth atomic shell (backscatterer), λ is the mean free path in Å, $\phi_j(k)$ is the phase shift (including the phase shift for each shell and the total central atom phase shift), σ_j is the Debye-Waller parameter of the jth atomic shell (variation of distances around the average R_j). The functions $F_j(k)$, λ and $\phi_j(k)$ were calculated with the ab initio code FEFF8.2. The obtained S_0^2 was fixed in the subsequent fitting. Since the composition of the sample is known, we fixed the interatomic distance (R) of CuO, Cu and Cu_2O in the fitting process. While the coordination number N, Debye-Waller factor σ^2, and the edge-energy shift ΔE_0 were allowed to run freely. P1 represents the percentage of CuO in the total content； P2 represents the percentage of Cu in the total content; P3 (P3=1-P1-P2) represents the percentage of Cu_2O in the total content.

Computational Method

Computational Method. Spin-polarized DFT calculations were performed with periodic super-cells under the generalized gradient approximation (GGA) using the Perdew-Burke-Ernzerhof (PBE) functional for exchange-correlation and the ultrasoft pseudopotentials for nuclei and core electrons. The Kohn-Sham orbitals were expanded in a plane-wave basis set with a kinetic energy cutoff of 30 Ry and the charge-density cutoff of 300 Ry. The Fermi-surface effects were treated by the smearing technique of Methfessel and Paxton, using a smearing parameter of 0.02 Ry. The models we use in this paper were hybrid structures with Cu and Cu_2O joint by respective (111) surfaces, with Cu_2O(111) located underneath 3 and 2 layered Cu(111) to simulate different content of Cu(I) (Fig. S25). For such hybrid structures, the lattice of Cu is remained to its optimized value while that of Cu_2O is compressed by 20%. To study the role of CeO_2, a cluster of CeO_2 was built on the surface (Fig. S25). All of the structure optimizations were proceeded at the supercells of 4x4's Cu(111), while the transition state calculations for CeO_2 associated structure were also done under 4x8's supercell. For all of the structures, only the first layer of the substrates was allowed to relax. The convergence criteria were set as 10^{-3} Ry/Bohr of Cartesian force components acting on each atom and 10^{-5} Ry of total energy. The Brillouin-zones were sampled with a 1×1×1 k-point mesh. The PWSCF codes

contained in the Quantum ESPRESSO distribution[3] were used to implement the calculations.

The transition state (TS) calculation involved the CO* protonation step to generate CHO* (RS3) on structures with and without CeO_2. Specifically, to get the saddle points and minimum energy paths (MEPs), we first used normal elastic band (NEB) method with 5 images, then interpolated with 2 images around the structure with highest energy achieved, and restarted the calculation with climbing image nudged method (CI-NEB).[4] The quasi-Newton method was employed to optimize the transition-state images from the NEB calculations until a maximum force less than 0.015 Ry/Å was achieved.

The details for composing the free energy diagram:

The reactions associated in this paper are

$$CO^*+CO^* \rightarrow {}^*CO^*CO \qquad (RS1)$$

$$CO^*+H_2O+e \rightarrow COH^*+OH^- \qquad (RS2)$$

$$CO^*+H_2O+e \rightarrow CHO^*+OH^- \qquad (RS3)$$

$$CHO^*+CHO^* \rightarrow {}^*CHO^*CHO \qquad (RS4)$$

$$COH^*+COH^* \rightarrow {}^*COH^*COH \qquad (RS5)$$

For proton donation associated reaction, the free energy were calculated via the computational hydrogen electrode method proposed by Norskov et al.[5] The adsorption energy for adsorbates X* is defined by

$$\Delta E_{X*} = E_{X*}^{DFT} - E_{*}^{DFT}$$

Where E_{X*}^{DFT} and E_{*}^{DFT} are the DFT based total energy of the structures with and without X* adsorption. The zero point energy for the adsorbates are not involved, which has been considered negligible based on the calculations in previous report.[6]

Supplementary Figure

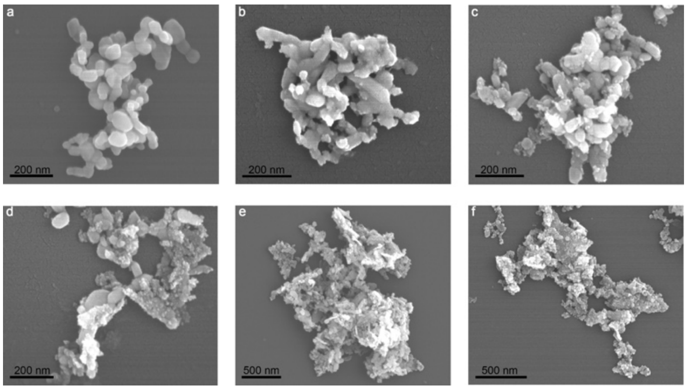

Fig. S1. (a-e) The SEM images of CC0, CC1, CC5, CC10, CC15, CC30.

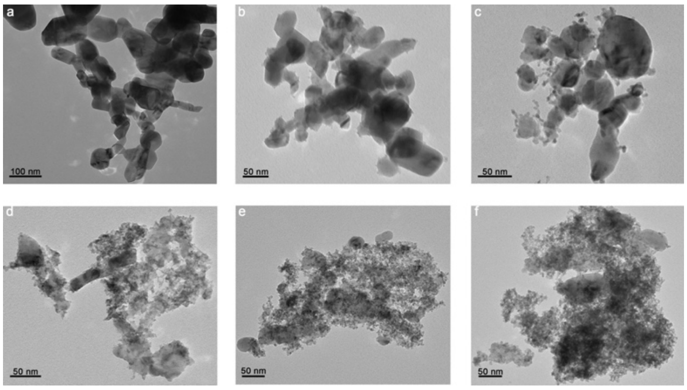

Fig. S2. (a-e) The TEM images of CC0, CC1, CC5, CC10, CC15, CC30.

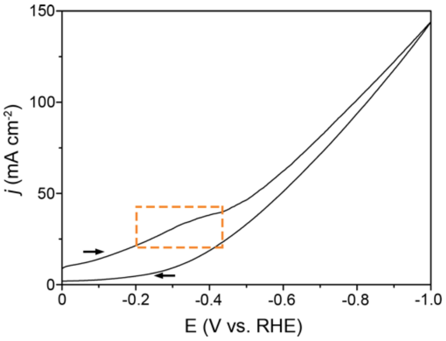

Fig. S3. The first cycle of CV on CC20 in 1 M KOH electrolyte in a flow cell during CO_2RR, and the orange column represents the reduction of CuO to Cu.

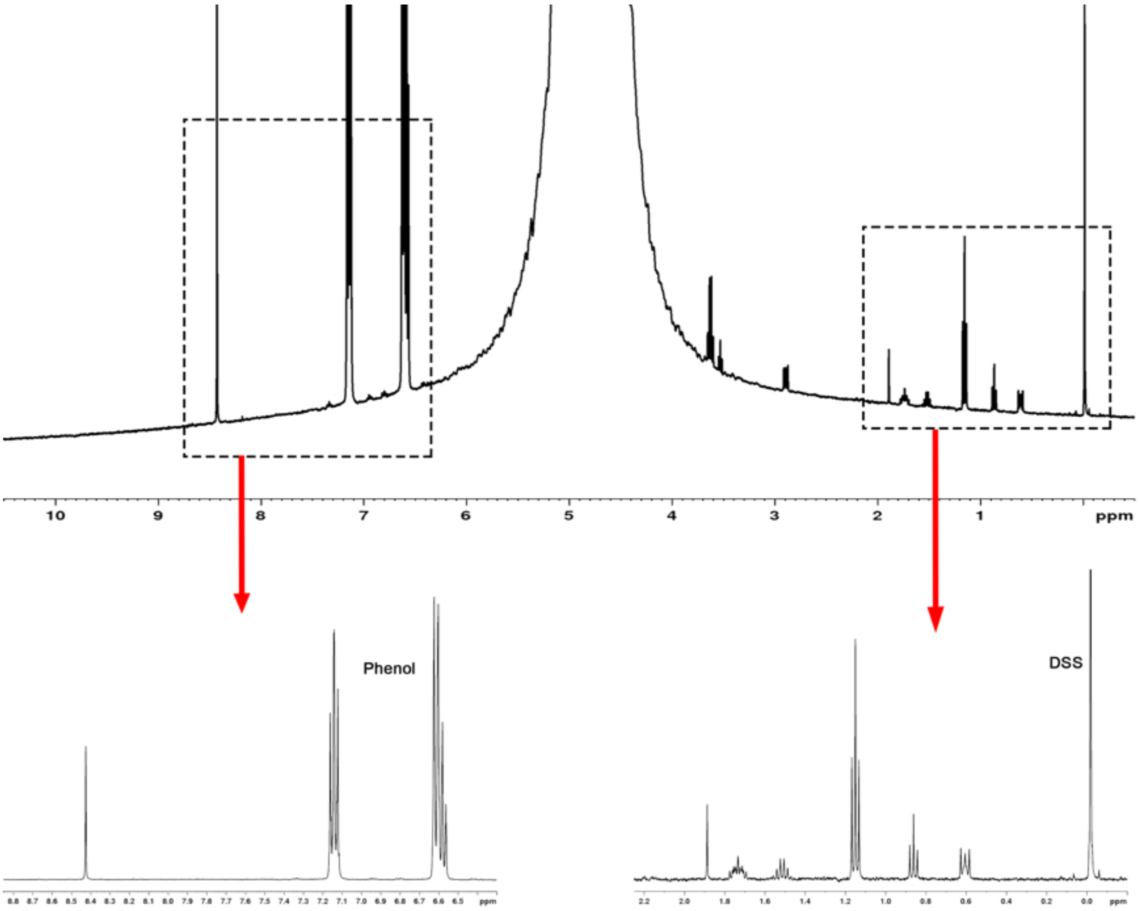

Fig. S4. A typical ^{1}H NMR spectrum of liquid products after electrolysis at -1.07 V (vs RHE) over CC20.

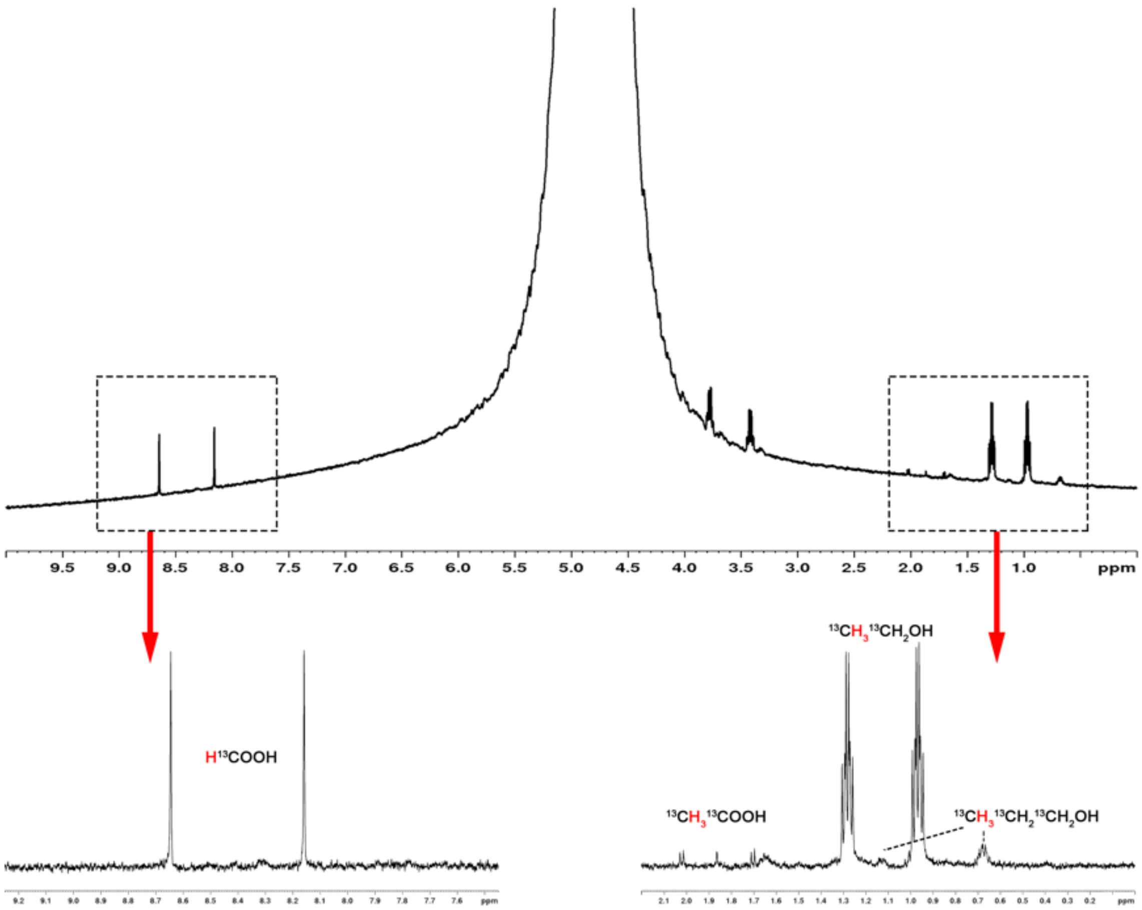

Fig. S5. The ^{1}H NMR plot of the liquid products after electrolysis at -1.07 V (vs RHE) using $^{13}CO_2$ as gas source.

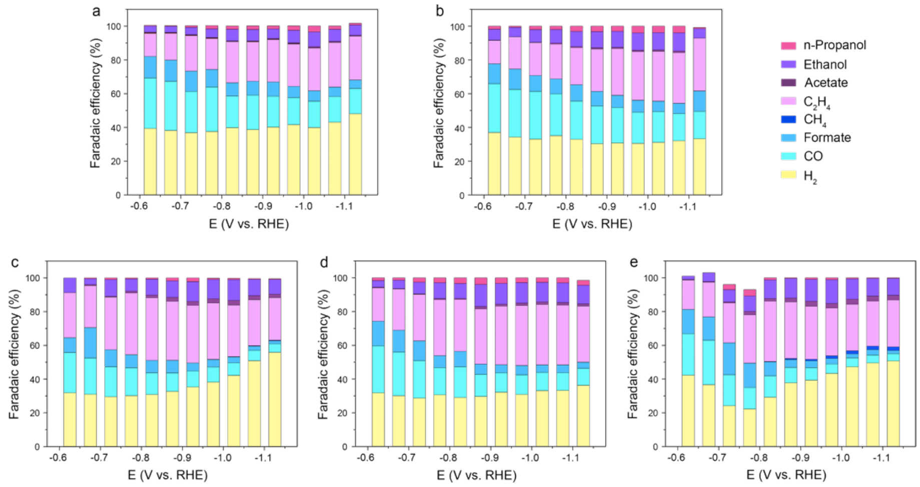

Fig. S6. (a-e) The FE of products for CC1, CC5, CC10, CC15, CC30 at various applied potentials in CO_2RR.

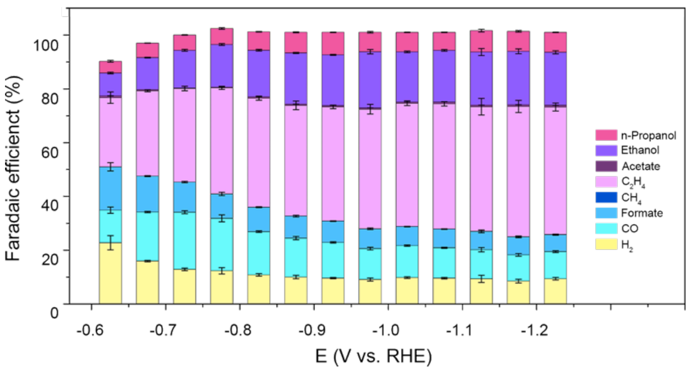

Fig. S7. The distribution of products over CC20 at various applied potentials in CO_2RR.

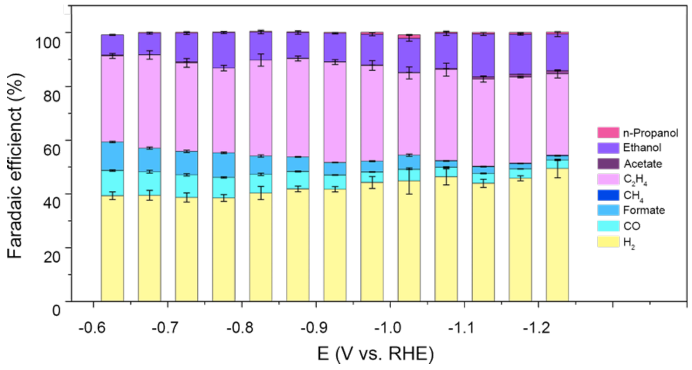

Fig. S8. The distribution of products over CC0 at various applied potentials in CO_2RR.

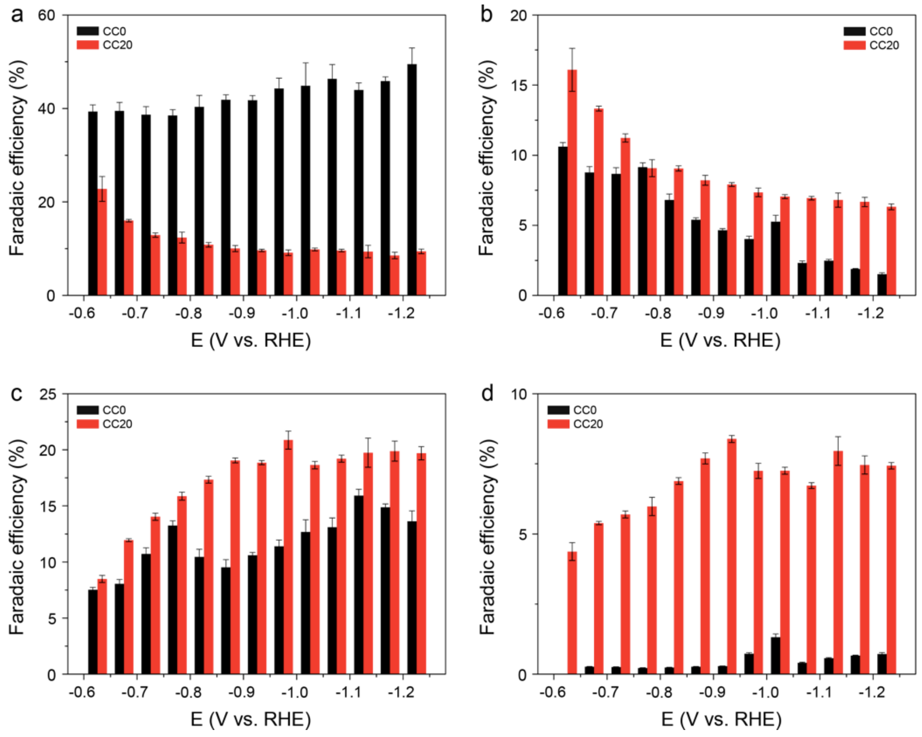

Fig. S9. The comparison for the FE of H_2 (a), formate (b), ethanol (c) and n-propanol (d) on CC0 and CC20 at various applied potentials in CO_2RR.

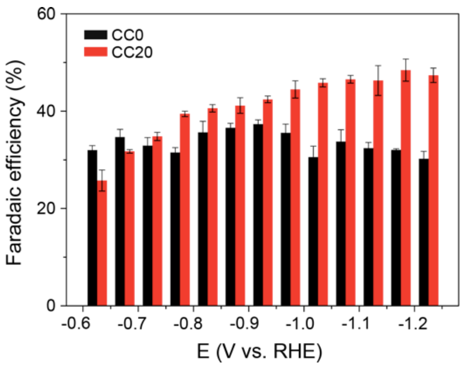

Fig. S10. The comparison for the FE of C_2H_4 on CC0 and CC20 at various applied potentials in CO_2RR.

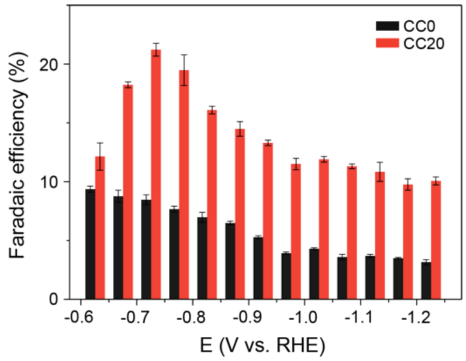

Fig. S11. The comparison for the FE of CO on CC0 and CC20 at various applied potentials in CO_2RR.

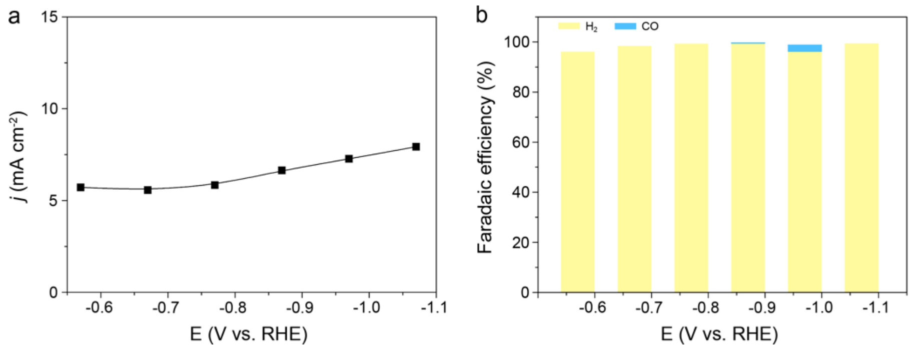

Fig. S12. (a) The current density and (b) faradaic efficiency of products on CeO_2 at various applied potentials in CO_2RR.

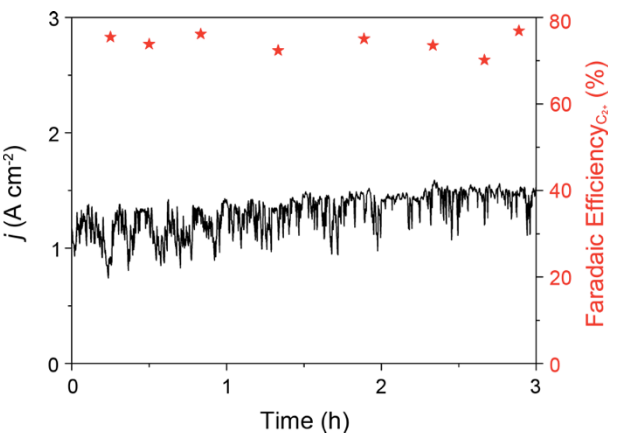

Fig. S13. The long-term stability test in 1 M KOH with the CO_2 flow rate as 20 sccm at -1.12 V (vs RHE) on CC20.

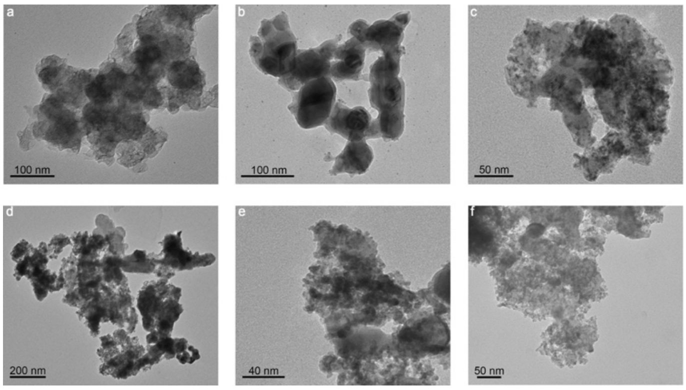

Fig. S14. (a-e) The TEM images of CC0, CC1, CC5, CC10, CC15, CC30 after the electrochemical test.

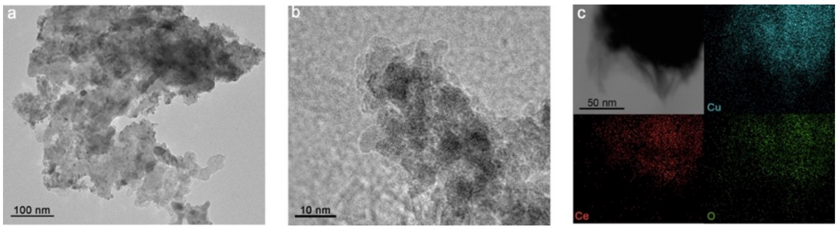

Fig. S15. (a) The TEM, (b) HR-TEM images and (c) EDS spectra of CC20 after the long-term stability test.

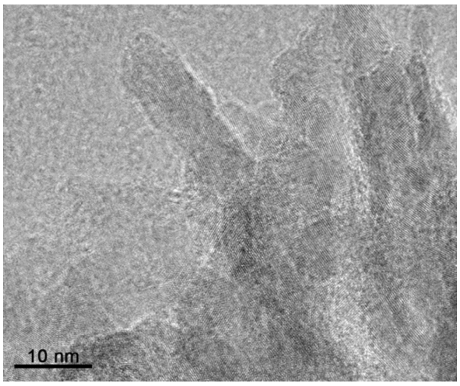

Fig. S16. The HR-TEM images of CC0 after the electrochemical test.

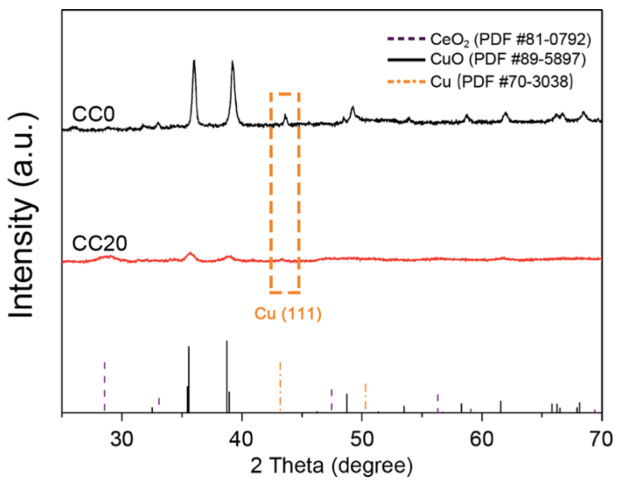

Fig. S17. The XRD patterns for CC0 and CC20 on PTFE membranes after CO_2RR, the orange column represents the corresponding peak of Cu(111).

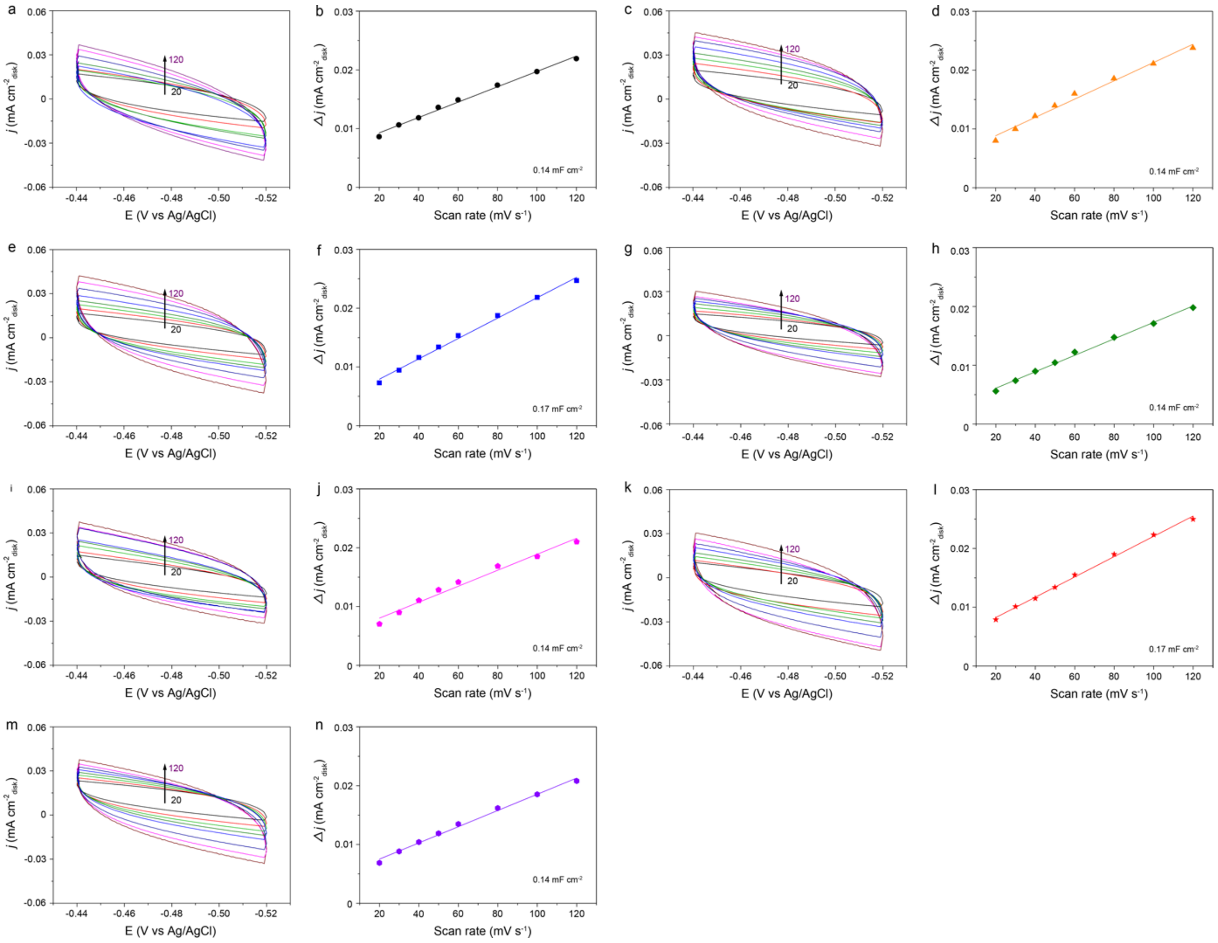

Fig. S18. (a, c, e, g, i, j, l) The cyclic voltammetry at various scan rates (20, 30, 40, 50, 60, 80, 100, 120 mV s^{-1}) over CC0, CC1, CC5, CC10, CC15, CC20, CC30; (b, d, f, h, k, m) the charging current density differences plotted against the scan rates over CC0, CC1, CC5, CC10, CC15, CC20, CC30.

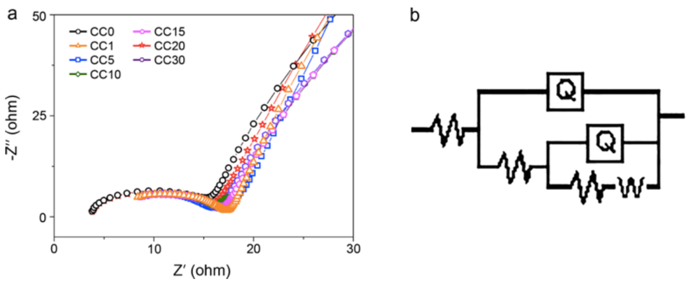

Fig. S19. Nyquist plots for different electrodes in CO_2-saturated 1 M KOH electrolyte.

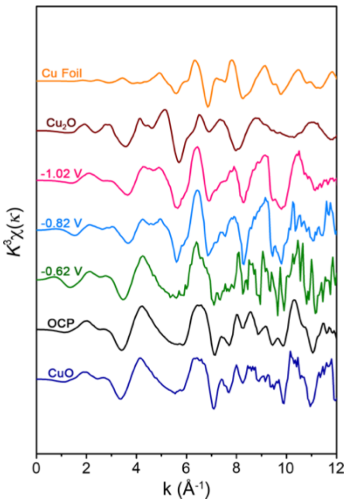

Fig. S20. *Operando* Cu K-edge extended XAFS oscillation function k^3w(k) at various applied potentials for CC20.

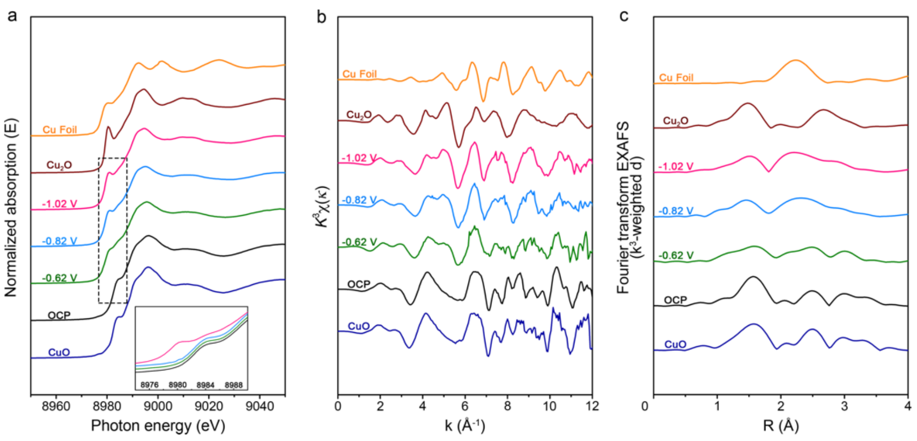

Fig. S21. (a) *Operando* XANES , (b) the corresponding extended XAFS oscillation function k^3w(k), and (c) Fourier transforms of k^3-weighted EXAFS data at Cu K-edge at various applied potentials over CC0.

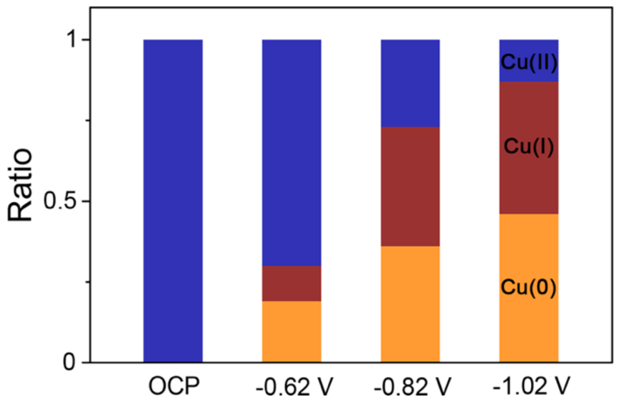

Fig. S22. The ratios of Cu species [Cu(0), Cu(I) and Cu(II)] at various applied potentials over CC20.

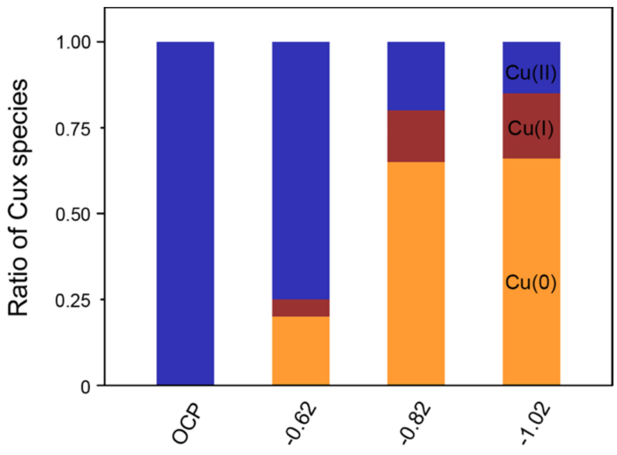

Fig. S23. The ratios of Cu species [Cu(0), Cu(I) and Cu(II)] at various applied potentials over CC0.

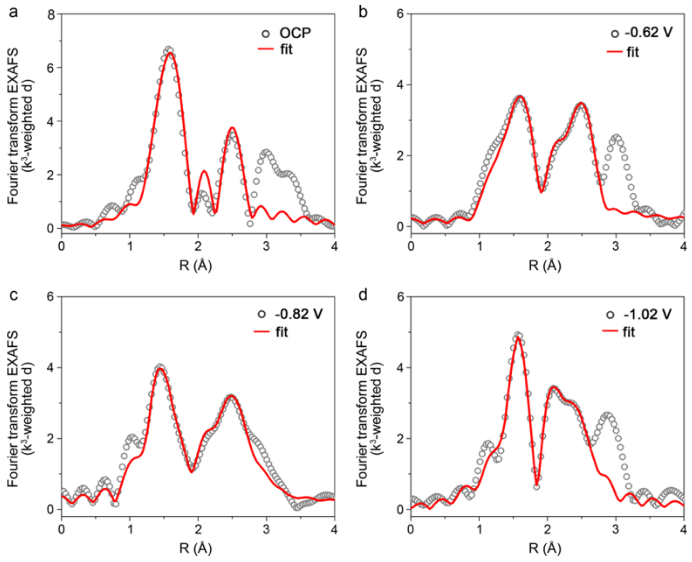

Fig. S24. The EXAFS data fitting results of CC0 at various applied potentials.

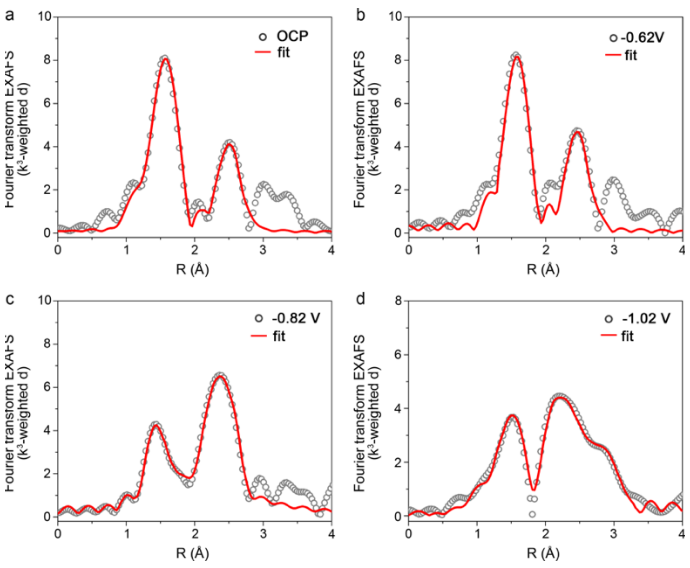

Fig. S25. The EXAFS data fitting results of CC20 at various applied potentials.

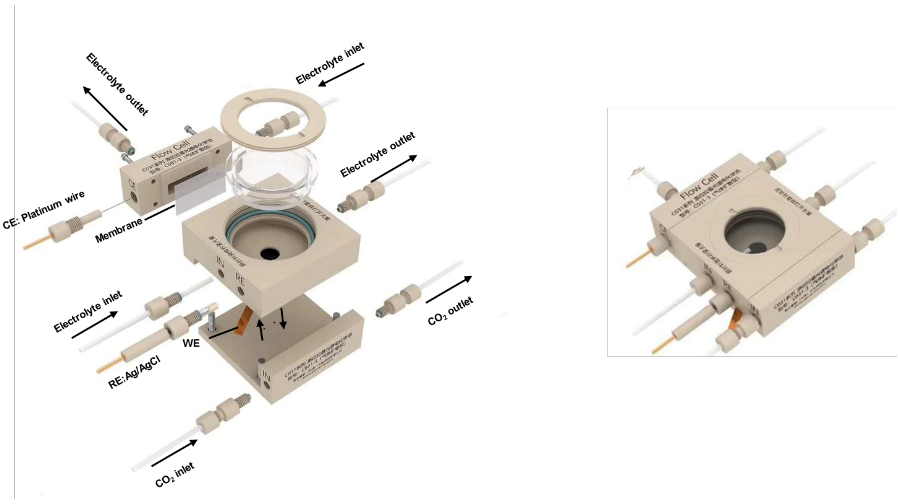

Fig. S26. *In-situ* electrochemical spectral cell for SERS test.

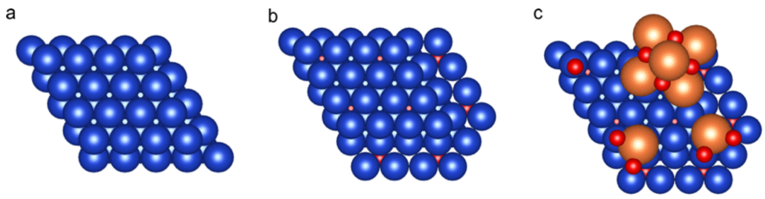

Fig. S27. (a) Top views of the Cu-M, Cu-L and CeO_2/Cu-L. Blue balls, yellow balls, red balls, gray balls and white balls stand for Cu, Ce, O, C and H, respectively.

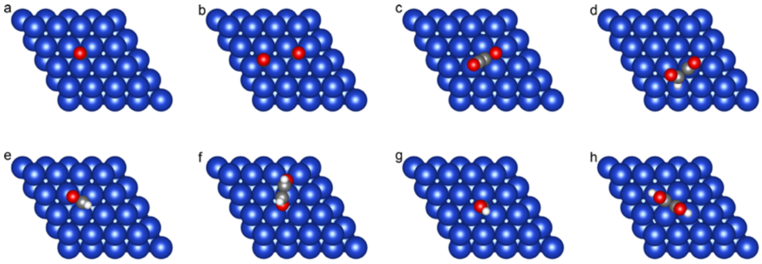

Fig. S28. Top view of (a) *CO, (b) *CO-*CO, (c) *O*CCO, (d) O*CCHO, (e) *CHO, (f) *OHCCHO*, (g) *COH, (h) *COH*COH on the Cu-M. Blue balls, red balls, gray balls and white balls stand for Cu, O, C and H, respectively.

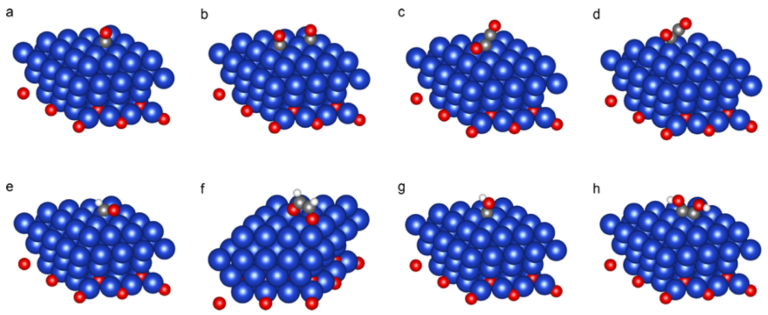

Fig. S29. Side view of (a) *CO, (b) *CO-*CO, (c) *O*CCO, (d) O*CCHO, (e) *CHO, (f) *OHCCHO*, (g) *COH, (h) *COH*COH on the Cu-M.

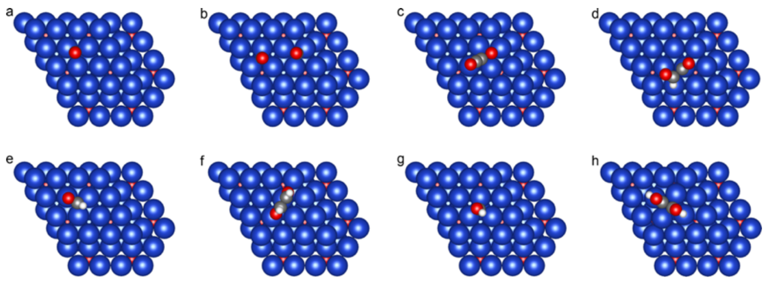

Fig. S30. Top view of (a) *CO, (b) *CO-*CO, (c) *O*CCO, (d) O*CCHO, (e) *CHO, (f) *OHCCHO*, (g) *COH, (h) *COH*COH on the Cu-L.

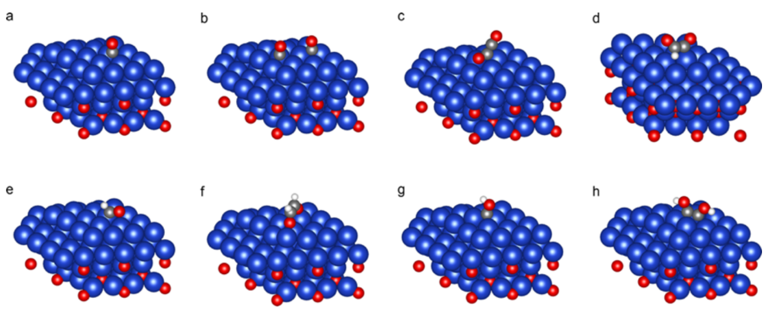

Fig. S31. Side view of (a) *CO, (b) *CO-*CO, (c) *O*CCO, (d) O*CCHO, (e) *CHO, (f) *OHCCHO*, (g) *COH, (h) *COH*COH on the Cu-L.

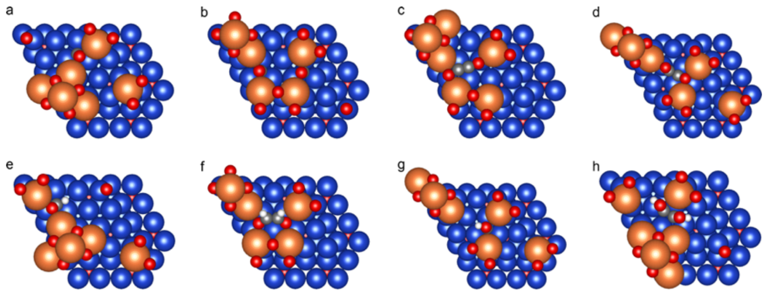

Fig. S32. Top view of (a) *CO, (b) *CO-*CO, (c) *OCCO*, (d) O*CCHO, (e) *CHO, (f) *OHCCHO*, (g) *COH, (h) *COH*COH on the CeO_2/Cu-L. Blue balls, yellow balls, red balls, gray balls and white balls stand for Cu, Ce, O, C and H, respectively.

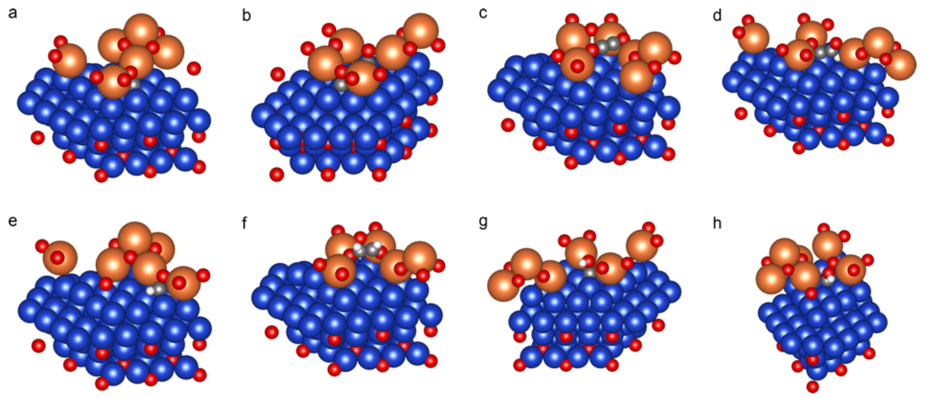

Fig. S33. Side view of (a) *CO, (b) *CO-*CO, (c) *OCCO*, (d) O*CCHO, (e) *CHO, (f) *OHCCHO*, (g) *COH, (h) *COH*COH on the CeO_2/Cu-L.

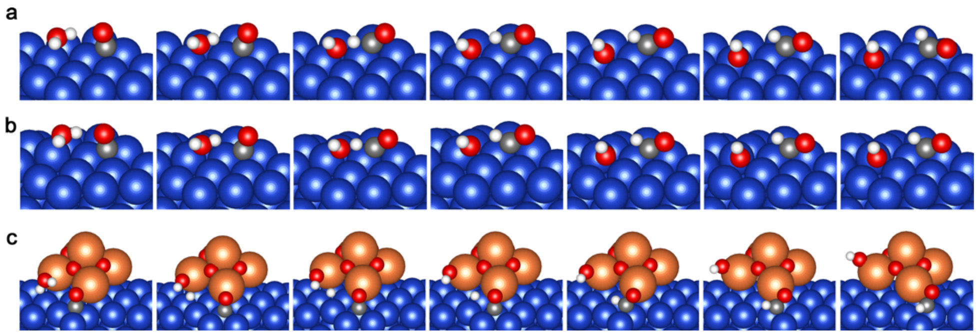

Fig. S34. (a, b, c) Side views of the key states in the hydrogenation of *CO into *CHO in the presence of H_2O* on Cu-M, Cu-L and CeO_2/Cu-L. Blue balls, yellow balls, red balls, gray balls and white balls stand for Cu, Ce, O, C and H, respectively.

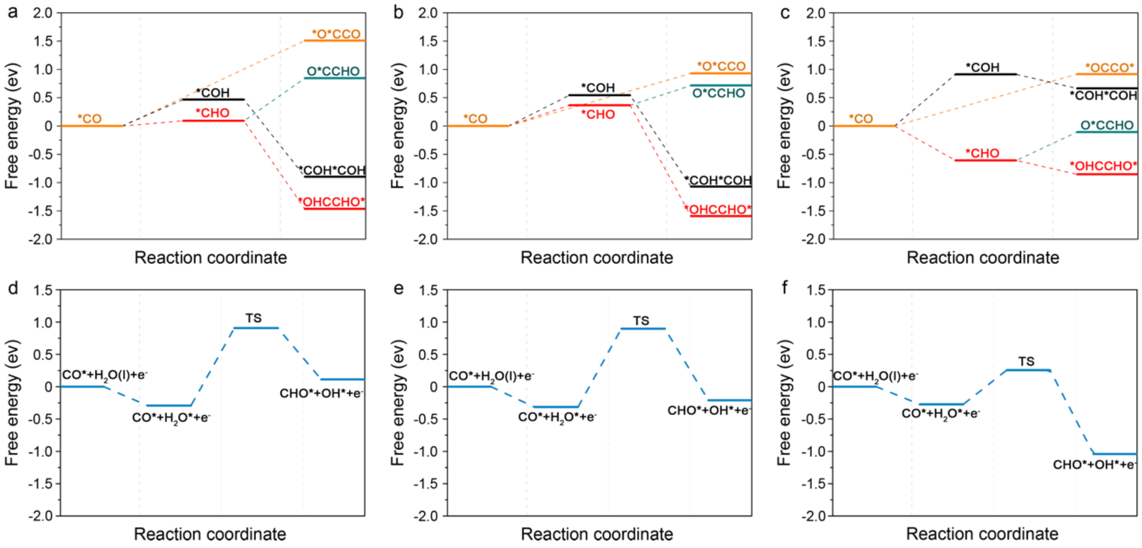

Fig. S35. (a, b, c) Reaction energy diagram for the CO_2RR to describe the possible C-C coupling step from *CO on Cu-M, Cu-L and CeO_2/Cu-L after applying a -0.5 V bias potential (vs RHE). (d, e, f) Reaction energy diagram for *CO hydrogenation to *COH on Cu-M, Cu-L and CeO_2/Cu-L after applying a -0.5 V bias potential (vs RHE).

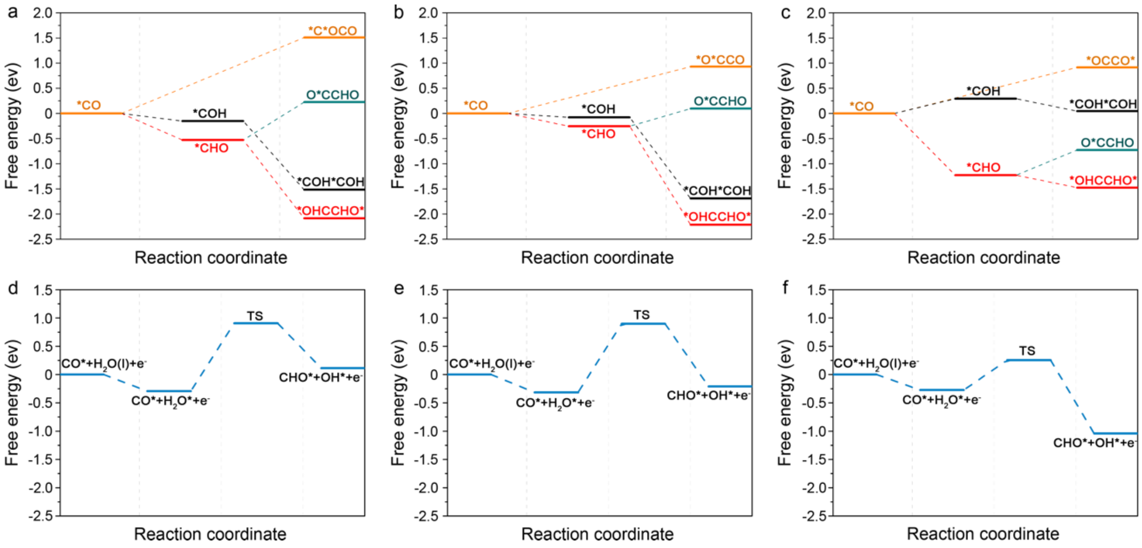

Fig. S36. (a, b, c) Reaction energy diagram for the CO_2RR to describe the possible C-C coupling step from *CO on Cu-M, Cu-L and CeO_2/Cu-L after applying a -1.12 V bias potential (vs RHE). (d, e, f) Reaction energy diagram for *CO hydrogenation to *COH on Cu-M, Cu-L and CeO_2/Cu-L after applying a -1.12 V bias potential (vs RHE).

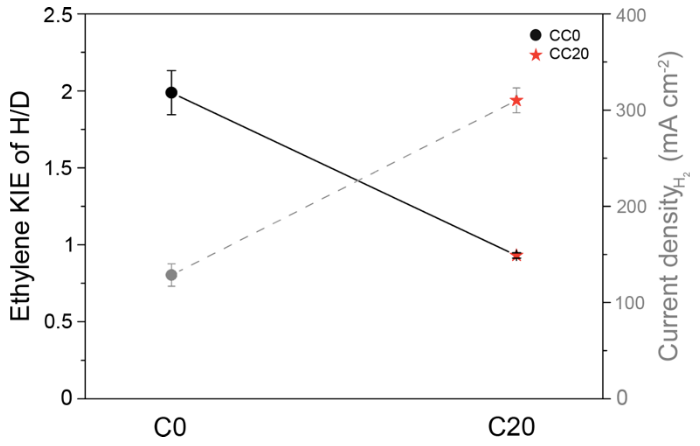

Fig. S37. KIEs of H/D in CO_2RR to ethylene in 1 M KOH (D_2O) with the CO_2 flow rate as 20 sccm and hydrogen evolution performance under a nitrogen atmosphere in 1 M KOH at -1.02 V versus RHE on CC20 and CC0.

Supplementary Tables

Table 1. The Comparison of C_{2+} products in CO_2RR on various Cu-based catalysts.

Samples	E (V) vs. RHE	FE_{C2+} (%)	j_{C2+} (mA cm^{-2})	References
CC20	-1.12	75.2	913.2	This work
NGQ/Cu-nr	-0.9	80.4	226.81	S2
Cu_KI	-1.09	72.58	29.03	S7
FeTPP[Cl]/Cu	-0.82	85	257.04	S8
Cu-CO_2	-0.71	90	519.3	S9
CuAg wire	-0.7	85	255	S10
F-Cu	-0.89	80	1280	S6
Nanoporous Cu	-0.67	62	404.86	S1
Dealloyed Cu	-1.8 ~ -2.1	75	450	S11
I-modified Cu	-0.9	80	31.2	S12
Reconstructed Cu	-1.8	77	346.5	S13
100-cycle Cu	-0.963	60.5	41	S14
OBC	-1	45	44.7	S15
Cu nanocubes	-0.8	60	44.4	S16
Multihollow Cu_2O	-0.61	75	267±13	S17
3D-CIBH	-0.91	91.7	1210	S18

Table 2. Structural parameters extracted from the EXAFS fitting for CC0. (S_0^2=0.85)

Sample	Scattering pair	P	CN	$\sigma^2(10^{-3}Å^2)$	ΔE_0(eV)	R factor
OCP	Cu-O(CuO)	P1=1.0	1.7	4.4	-3.4	0.01
	Cu-Cu(CuO)		4.2	4.6	3.1	
	Cu-Cu (Cu foil)	P2=0	/	/	/	/
	Cu-O（Cu_2O）	P3=0	/	/	/	/
	Cu-Cu（Cu_2O）		/	/	/	/
-0.62 V	Cu-O(CuO)	P1=0.75	1.7	4.9	-3.4	0.01
	Cu-Cu(CuO)		4.1	5.2	3.1	
	Cu-Cu (Cu foil)	P2=0.15	10	5.3	4.0	0.02
	Cu-O（Cu_2O）	P3=0.05	2.3	5.2	-3.6	0.01
	Cu-Cu（Cu_2O）		2.1	5.3	4.2	
-0.82 V	Cu-O(CuO)	P1=0.2	1.6	5.2	-3.4	0.01
	Cu-Cu(CuO)		3.9	5.3	3.1	
	Cu-Cu (Cu foil)	P2=0.65	11.5	5.6	4.0	0.01
	Cu-O（Cu_2O）	P3=0.15	2.4	5.5	-3.6	0.01
	Cu-O（Cu_2O）		2.2	5.7	4.2	
-1.02 V	Cu-O(CuO)	P1=0.15	1.7	5.5	-3.4	0.01
	Cu-Cu(CuO)		4.0	5.9	3.1	
	Cu-Cu (Cu foil)	P2=0.66	11.2	7.0	4.0	0.02
	Cu-O（Cu_2O）	P3=0.21	3.0	6.1	-3.6	0.01
	Cu-O（Cu_2O）		3.0	6.5	4.2	

S_0^2 is the amplitude reduction factor; CN is the coordination number; R is interatomic distance (the bond length between central atoms and surrounding coordination atoms); σ^2 is Debye-Waller factor (a measure of thermal and static disorder in absorber-scatterer distances); ΔE_0 is edge-energy shift (the difference between the zero kinetic energy value of the sample and that of the theoretical model). R factor is used to value the goodness of the fitting.

Table 3. Structural parameters extracted from the EXAFS fitting for CC20. (S_0^2=0.85)

Sample	Scattering pair	P	CN	$\sigma^2(10^{-3}Å^2)$	ΔE_0(eV)	R factor
OCP	Cu-O(CuO)	P1=1	1.8	4.4	-3.4	0.01
	Cu-Cu(CuO)		4.3	4.6	3.1	
	Cu-Cu (Cu foil)	P2=0	/	/	/	/
	Cu-O（Cu_2O）	P3=0	/	/	/	/
	Cu-Cu（Cu_2O）		/	/	/	/
-0.62 V	Cu-O(CuO)	P1=0.7	1.7	4.9	-3.4	0.01
	Cu-Cu(CuO)		4.3	5.3	3.1	
	Cu-Cu (Cu foil)	P2=0.19	10	5.1	4.0	0.02
	Cu-O（Cu_2O）	P3=0.11	2.4	5.2	-3.4	0.01
	Cu-Cu（Cu_2O）		2.1	5.3	4.2	
-0.82 V	Cu-O(CuO)	P1=0.27	1.8	5.0	-3.4	0.01
	Cu-Cu(CuO)		4.0	5.4	3.1	
	Cu-Cu (Cu foil)	P2=0.37	10	6.3	4.1	0.02
	Cu-O（Cu_2O）	P3=0.36	3.0	5.8	-3.4	0.01
	Cu-O（Cu_2O）		2.8	6.1	4.2	
-1.02 V	Cu-O(CuO)	P1=0.13	1.7	5.2	-3.4	0.01
	Cu-Cu(CuO)		4.0	5.7	3.1	
	Cu-Cu (Cu foil)	P2=0.41	10	6.8	4.1	0.01
	Cu-O（Cu_2O）	P3=0.46	3.5	6.1	-3.4	0.01
	Cu-O（Cu_2O）		3.2	6.5	4.1	

S_0^2 is the amplitude reduction factor; CN is the coordination number; R is interatomic distance (the bond length between central atoms and surrounding coordination atoms); σ^2 is Debye-Waller factor (a measure of thermal and static disorder in absorber-scatterer distances); ΔE_0 is edge-energy shift (the difference between the zero kinetic energy value of the sample and that of the theoretical model). R factor is used to value the goodness of the fitting.

Notes and References

1 J. J. Lv, M. Jouny, W. Luc, W. L. Zhu, J. J. Zhu and F. Jiao, *Adv. Mater.*, 2018, **30**, e180311.

2 C. J. Chen, X. P. Yan, S. J. Liu, Y. H. Wu, Q. Wan, X. F. Sun, Q. G. Zhu, H. Z. Liu, J. Ma, L. R. Zheng, H. H. Wu and B. X. Han, *Angew. Chem. Int. Ed.*, 2020, **59**, 16459-16464.

3 P. Giannozzi, S. Baroni, N. Bonini, M. Calandra, R. Car, C. Cavazzoni, D. Ceresoli, G. L. Chiarotti, M. Cococcioni, I. Dabo, A. Dal Corso, S. de Gironcoli, S. Fabris, G. Fratesi, R. Gebauer, U. Gerstmann, C. Gougoussis, A. Kokalj, M. Lazzeri, L. Martin-Samos, N. Marzari, F. Mauri, R. Mazzarello, S. Paolini, A. Pasquarello, L. Paulatto, C. Sbraccia, S. Scandolo, G. Sclauzero, A. P. Seitsonen, A. Smogunov, P. Umari and R. M. Wentzcovitch, *J Phys-Condens Mat*, 2009, **21**, 395502.

4 G. Henkelman, B. P. Uberuaga and H. Jonsson, *J. Chem. Phys.*, 2000, **113**, 9901-9904.

5 J. K. Norskov, J. Rossmeisl, A. Logadottir, L. Lindqvist, J. R. Kitchin, T. Bligaard and H. Jonsson, *J. Phys. Chem. B*, 2004, **108**, 17886-17892.

6 W. C. Ma, S. J. Xie, T. T. Liu, Q. Y. Fan, J. Y. Ye, F. F. Sun, Z. Jiang, Q. H. Zhang, J. Cheng and Y. Wang, *Nat. Catal.*, 2020, **3**, 478-487.

7 T. Kim and G. T. R. Palmore, *Nat. Commun.*, 2020, **11**, 3622.

8 F. W. Li, Y. G. C. Li, Z. Y. Wang, J. Li, D. H. Nam, Y. Lum, M. C. Luo, X. Wang, A. Ozden, S. F. Hung, B. Chen, Y. H. Wang, J. Wicks, Y. Xu, Y. L. Li, C. M. Gabardo, C. T. Dinh, Y. Wang, T. T. Zhuang, D. Sinton and E. H. Sargent, *Nat. Catal.*, 2020, **3**, 75-82.

9 Y. H. Wang, Z. Y. Wang, C. T. Dinh, J. Li, A. Ozden, M. G. Kibria, A. Seifitokaldani, C. S. Tan, C. M. Gabardo, M. C. Luo, H. Zhou, F. W. Li, Y. Lum, C. McCallum, Y. Xu, M. X. Liu, A. Proppe, A. Johnston, P. Todorovic, T. T. Zhuang, D. Sinton, S. O. Kelley and E. H. Sargent, *Nat. Catal.*, 2020, **3**, 98-106.

10 T. T. H. Hoang, S. Verma, S. C. Ma, T. T. Fister, J. Timoshenko, A. I. Frenkel, P. J. A. Kenis and A. A. Gewirth, *J. Am. Chem. Soc.*, 2018, **140**, 5791-5797.

11 M. Zhong, K. Tran, Y. M. Min, C. H. Wang, Z. Y. Wang, C. T. Dinh, P. De Luna, Z. Q. Yu, A. S. Rasouli, P. Brodersen, S. Sun, O. Voznyy, C. S. Tan, M. Askerka, F. L. Che, M. Liu, A. Seifitokaldani, Y. J. Pang, S. C. Lo, A. Ip, Z. Ulissi and E. H. Sargent, *Nature*, 2020, **581**, 178-183.

12 D. F. Gao, I. Sinev, F. Scholten, R. M. Aran-Ais, N. J. Divins, K. Kvashnina, J. Timoshenko and B. Roldan Cuenya, *Angew. Chem. Int. Ed.*, 2019, **58**, 17047-17053.

13 M. G. Kibria, C. T. Dinh, A. Seifitokaldani, P. De Luna, T. Burdyny, R. Quintero-Bermudez, M. B. Ross, O. S. Bushuyev, F. P. G. de Arguer, P. D. Yang, D. Sinton and E. H. Sargen, *Adv. Mater.*, 2018, **30**, e1804867.

14 K. Jiang, R. B. Sandberg, A. J. Akey, X. Y. Liu, D. C. Bell, J. K. Norskov, K. R. Chan and H. T. Wang, *Nat. Catal.*, 2018, **1**, 111-119.

15 W. Zhang, C. Q. Huang, Q. Xiao, L. Yu, L. Shuai, P. F. An, J. Zhang, M. Qiu, Z. F. Ren and Y. Yu, *J. Am. Chem. Soc.*, 2020, **142**, 11417-11427.

16 Y. X. Wang, H. Shen, K. J. T. Livi, D. Raciti, H. Zong, J. Gregg, M. Onadeko, Y. D. Wan, A. Watson and C. Wang, *Nano Letters*, 2019, **19**, 8461-8468.

17 P. P. Yang, X. L. Zhang, F. Y. Gao, Y. R. Zheng, Z. Z. Niu, X. X. Yu, R. Liu, Z. Z. Wu, S. Qin, L. P. Chi, Y. Duan, T. Ma, X. S. Zheng, J. F. Zhu, H. J. Wang, M. R. Gao and S. H. Yu, *J. Am. Chem. Soc.*, 2020, **142**, 6400-6408.

18 F. P. G. de Arquer, C. T. Dinh, A. Ozden, J. Wicks, C. McCallum, A. R. Kirmani, D. H. Nam, C. Gabardo, A. Seifitokaldani, X. Wang, Y. G. C. Li, F. W. Li, J. Edwards, L. J. Richter, S. J. Thorpe, D. Sinton and E. H. Sargent, *Science*, 2020, **367**, 661-666.
